# Faecal Microbiota of Forage-Fed Horses in New Zealand and the Population Dynamics of Microbial Communities following Dietary Change

**DOI:** 10.1371/journal.pone.0112846

**Published:** 2014-11-10

**Authors:** Karlette A. Fernandes, Sandra Kittelmann, Christopher W. Rogers, Erica K. Gee, Charlotte F. Bolwell, Emma N. Bermingham, David G. Thomas

**Affiliations:** 1 Institute of Veterinary, Animal, and Biomedical Sciences, College of Sciences, Massey University, Palmerston North 4442, New Zealand; 2 AgResearch Ltd, Grasslands Research Centre, Palmerston North 4442, New Zealand; Agriculture and Agri-Food Canada, Canada

## Abstract

The effects of abrupt dietary transition on the faecal microbiota of forage-fed horses over a 3-week period were investigated. Yearling Thoroughbred fillies reared as a cohort were exclusively fed on either an ensiled conserved forage-grain diet (“Group A”; n = 6) or pasture (“Group B”; n = 6) for three weeks prior to the study. After the Day 0 faecal samples were collected, horses of Group A were abruptly transitioned to pasture. Both groups continued to graze similar pasture for three weeks, with faecal samples collected at 4-day intervals. DNA was isolated from the faeces and microbial 16S and 18S rRNA gene amplicons were generated and analysed by pyrosequencing. The faecal bacterial communities of both groups of horses were highly diverse (Simpson’s index of diversity >0.8), with differences between the two groups on Day 0 (*P*<0.017 adjusted for multiple comparisons). There were differences between Groups A and B in the relative abundances of four genera, BF311 (family Bacteroidaceae; *P* = 0.003), CF231 (family Paraprevotellaceae; *P* = 0.004), and currently unclassified members within the order Clostridiales (*P* = 0.003) and within the family Lachnospiraceae (*P* = 0.006). The bacterial community of Group A horses became similar to Group B within four days of feeding on pasture, whereas the structure of the archaeal community remained constant pre- and post-dietary change. The community structure of the faecal microbiota (bacteria, archaea and ciliate protozoa) of pasture-fed horses was also identified. The initial differences observed appeared to be linked to recent dietary history, with the bacterial community of the forage-fed horses responding rapidly to abrupt dietary change.

## Introduction

The horse is a cursorial grazer with the ability to efficiently utilise high-fibre grass and other forages [1], [2]. The majority of ingested plant fibre is comprised of structural carbohydrates such as cellulose, hemicellulose, and lignin, which cannot be digested by host enzymes in the foregut. As a result, the undigested plant material reaches the hindgut where breakdown of cellulose and hemicellulose occurs through the process of microbial fermentation, generating energy-yielding products such as volatile fatty acids (VFAs) [3], [4]. It is estimated that forage-fed horses may obtain 50–70% of their energy requirements from VFAs [5].

Several species of microbes including bacteria, archaea and eukarya (protozoa and fungi) inhabit the equine gastrointestinal tract [6], [7]. However, the bacterial community, which represents the major proportion of the hindgut microbiota, has been the focus of much of the published literature and has predominantly been investigated using culture-based techniques [8], [9], [10]. Other molecular techniques independent of culture, such as polymerase chain reaction (PCR), denaturing gradient gel electrophoresis (DGGE), fluorescent in-situ hybridization (FISH) and terminal restriction fragment length polymorphism (TRFLP), have also been used [8], [11], [12]. More recently, a few investigations have used metagenomics involving high-throughput next generation sequencing (pyrosequencing of 16S and 18S rRNA gene amplicons) to explore the equine hindgut microbiome (eHGM) *in vivo* [13], [14], [15]. However, there are still gaps in the current knowledge on the composition and the function of the microbial communities that inhabit the equine hindgut. This may be due to the difficulty in culturing anaerobic microorganisms and comparing results from studies that have used different experimental designs, sequencing methodologies and phylogenetic and statistical data analyses [8].

Because of the need to fistulate the horse to obtain hindgut samples, the majority of the work on the eHGM has focused on the use of material obtained from faecal samples [8]. From the limited comparative work conducted on the bacterial community of the equine hindgut and faeces, there appears to be good agreement between the microbiomes of the colon and faeces [15], [16].

The population of hindgut microbiota is sensitive to changes occurring in the gastrointestinal environment, with variations in the bacterial community structure reported in response to the composition of a diet, passage rate of digesta, and level of pre-caecal starch digestion [17], [18]. Abrupt dietary changes, particularly the availability of fermentable starch and water-soluble carbohydrates, can perturb the populations of microbes in the hindgut and can lead to digestive and metabolic disorders such as hindgut acidosis, colic and laminitis [6], [19], [20]. Forage-only diets promote greater microbial stability, evident by the lower microbial counts and relative abundances of specific lactic acid producing bacteria [11]. However, the effects of changes in forage-based diets on the bacterial population in the hindgut or faeces may be dependent on the type of horse (breed), composition of the diet and management practices [12], [21], [22].

Culture-based techniques have identified that faecal colony forming units of *Streptococcus* spp. and *Lactobacillus* spp. increase significantly from pasture baseline values within six days of an acute dietary transition to a grain-based diet, with numbers subsequently decreasing when the horses were returned back to grazing on pasture [23]. Similarly, an abrupt change from feeding 100% hay diets to a combination of hay and concentrates was reported to produce changes in the hindgut bacterial populations within 5 hours (caecum) and 29 hours (colon), with greater changes occurring in the colon [24]. More recently, an *in vitro* study suggested that bacterial communities isolated from horse faeces responded to a carbohydrate substrate within 12 hours, with significant changes in relative abundances of bacteria over a 48-hour period [25]. The above findings indicate that changes in the bacterial community occur rapidly within the hindgut, and may be evident in the faeces within a few days, depending on the type of diet and the transit time through the intestinal tract.

In New Zealand, equine pastures are predominantly comprised of a perennial ryegrass and white clover mix, with lesser quantities of other grasses and legumes, and many horses are kept or reared on pasture all year round [26], [27], [28]. The composition of microbiota in the faeces of New Zealand pasture-fed horses, or the effects of an abrupt dietary change from forage-grain diets to pasture on the hindgut or faecal microbiota, are currently unknown. The aims of the current study were to: 1) describe and compare the faecal microbiota of horses grazing on pasture to those fed exclusively on an ensiled conserved forage-grain diet in loose-boxes; and 2) investigate the changes in the relative abundance of faecal microbiota due to abrupt dietary change from an ensiled conserved forage-grain diet to pasture over a period of three weeks.

## Materials and Methods

### Ethics statement

The use of animals, including welfare, husbandry, experimental procedures, and collection of the faecal samples for this study, was approved by the Massey University Animal Ethics Committee (MUAEC), Massey University, Palmerston North, New Zealand (Protocol number 12–51).

### Experimental design and sample collection

Twelve yearling Thoroughbred fillies (mean age ± standard deviation [SD], 396±22 days) born and reared as a cohort on a commercial Thoroughbred stud farm (Palmerston North, Manawatu, New Zealand) were enrolled in the study during the spring of 2012 (November). Ten of the yearlings were sired by the same stallion. The general health and history of feeding management were recorded, and included daily observations by the stud master for any signs of illness or disease, weekly measurements of height, weight and body condition scores, anthelminthic treatments, and veterinary check-ups. The yearlings were in good health and had a median body condition score of six (Interquartile range [IQR] 5–6) on a 9-point scale [29], and a mean height and weight of 146.3±2.8 cm and 348.3±21.7 kg, respectively.

The 12 yearling horses were randomly divided into two treatment groups. For 21 days prior to Day 0, horses in Group A (n = 6) were kept in loose-boxes (4×4 m) lined with rubber matting, and were fed exclusively on a commercial ensiled conserved forage-grain-based ration (Diet F - FiberSure, Fiber Fresh Feeds Ltd., Reporoa, New Zealand), as part of a voluntary feed intake and digestibility study [30]. Diet F comprised of ensiled chaffed lucerne (*Medicago sativa*; alfalfa grass; 65%), cracked maize (*Zea mays*) grain (35%) and a vitamin and mineral premix with molasses (5%); the nutrient analysis of the diet is given in Table S1. The loose-boxes were arranged in a single row, the lower half of the internal walls were made of wood and the upper half made of wire mesh, allowing visual contact between all horses in the loose-boxes and the adjacent yard. The horses were turned-out for exercise in pairs, in a compact-earth yard adjacent to the loose-boxes, for 30 minutes twice a day [30]. Horses in Group B were kept in a paddock and were provided *ad libitum* pasture (Diet P; a standard New Zealand ryegrass-clover pasture comprised of ∼80–95% perennial ryegrass (*Lolium perenne*) and ∼5–20% white clover (*Trifolium repens*) [27]) for 21 days prior to Day 0. After the Day 0 faecal samples were collected, horses in Group A were abruptly transitioned to feeding on pasture (Diet P). Both groups of horses continued to graze on pasture for the next three weeks (21 days), during which the horses were kept in separate 1.5–2.0 hectare paddocks on the same property, containing pasture of similar herbage mass (pasture cover of 1600–2000 kg DM/ha/year) and nutrient content (Table S1).

Faecal samples were collected from all yearlings between 0900 and 1200 hours on Day 0, and subsequently at 4-day intervals over a period of 21 days (giving a total of 72 samples). The samples were collected within two minutes of defecation, using a forceps to collect representative faecal samples with minimal environmental contamination. These were immediately transferred into 3 ml polyethylene cryovials (Ray Lab Ltd., Auckland, New Zealand), and snap frozen in liquid nitrogen. The faecal samples were stored in a portable canister containing liquid nitrogen and transferred to a −80°C freezer within four hours of collection and stored until laboratory analysis.

### DNA extraction, PCR amplification of target genes and pyrosequencing

Nucleic acids were extracted from 100 mg of faeces by disrupting the cells by a combined bead-beating and phenol-chloroform-isoamyl alcohol (25∶24∶1; vol:vol:vol) treatment and subsequent precipitation of proteins with chloroform [31]. DNA was precipitated from the aqueous phase with two volumes of 30% (wt:vol) polyethylene glycol, washed with 70% (vol:vol) ice-cold ethanol, dried and eluted in 50 µl of elution buffer (EB; 10 mM Tris, pH 8.5 with HCl). Extracted DNA was quantified using the NanoDrop ND-1000 UV-Vis Spectrophotometer (NanoDrop Technologies, Wilmington, DE, USA) and normalized to 40 ng/µl.

Polymerase chain reaction (PCR) amplification of the bacterial 16S rRNA genes (V1–V3 regions), archaeal 16S rRNA genes (V6–V8 regions), and ciliate protozoal 18S rRNA genes (V5–V8 regions) were carried out as described previously, using universal primers (Ba515Rmod1, Ar915aF and Reg1302R) for the three groups of microorganisms [32]. All primers contained the 454 Life Science (Branford, CT, USA) adaptors A (5′-CCA TCT CAT CCC TGC GTG TCT CCG ACT CAG-3′) or B (5′-CCT ATC CCC TGT GTG CCT TGG CAG TCT CAG-3′) for Titanium sequencing, and a unique 12-base error-correcting barcode was attached to adaptor A for sample identification [33], [34]. A PCR master mix of 76 µl was prepared for each DNA sample (per microbial group), as previously described [32]. An aliquot of 19 µl was transferred to serve as a no-template negative control. The remaining 57 µl of reaction mix were spiked with 10–40 ng of DNA contained in 3 µl of water, and then divided into three aliquots of 20 µl each. Amplification was performed in a Mastercycler proS (Eppendorf, Hamburg, Germany) using a previously described protocol [32]. Triplicate PCR products were pooled, and correct sizes of PCR products and signal absence from the negative controls were verified by agarose gel electrophoresis.

Following quantification of PCR products using the Quant-iT dsDNA BR assay kit (Invitrogen, Carlsbad, CA, USA) and a fluorometer (BioTek Instruments, Winooski, VT, USA), amplicons of the same target gene and region were pooled into three separate pools and loaded onto a 1%-agarose gel (wt:vol) prepared with 1× TAE buffer (40 mM Tris, 20 mM acetic acid, 1 mM EDTA, pH 8 with NaOH). Bands were visualised under blue light transillumination, excised, and DNA purified from the gel slices with the QIAquick gel extraction kit (Qiagen, Hilden, Germany). Gel-purified amplicon pools were quantified in triplicate with the Quant-iT dsDNA HS assay kit (Invitrogen). The three amplicon pools were normalized to contain 1×10^9^ copies µl^−1^ and subsequently mixed at a ratio of 5∶1∶1 (Bacteria, Archaea, Protozoa) [32]. This final pool was sent to MWG Eurofins (Ebersberg, Germany) for Titanium pyrosequencing on a 454 Life Sciences Genome Sequencer FLX machine (454 Life Sciences, Branford, CT, USA).

### Phylogenetic analysis of pyrosequencing reads

Samples were processed and analysed using the software QIIME (Quantitative Insights Into Microbial Ecology) v1.5 [35]. Sequence reads were assigned to corresponding samples by examining the 12-nucleotide error-correcting Golay barcodes using the split_libraries.py script in QIIME. The split_libraries.py script (with default settings) selected sequences that had a minimum average quality score of 25, a maximum of six ambiguous bases with no allowance for mismatches in primer sequence, a maximum homopolymer run length of six and a maximum sequence length of 1000 bp. All sequences that did not meet the quality-filtering criteria were excluded from downstream analysis. For bacteria and archaea, only sequences that were ≥400 bp in length (including primers, option -l), and in which both the forward and reverse primers were detected, were selected for further analyses. The primers were subsequently removed (option -z truncate_remove), and this option allowed only high-quality sequences to be retained, in which the reverse primer sequence was unambiguously detected. The remaining sequences that did not meet the quality criteria were removed from the bacterial and archaeal sequence libraries. Bacterial and archaeal 16S rRNA gene sequence data were denoised using Acacia [36] and chimera checked using the blast fragment method in QIIME [35], [37] against the Greengenes database (gg_13_5/gg_13_5.fasta [38]). All sequences that did not meet the quality criteria, and those identified as pyrosequencing noise or potential chimeras, were removed from the bacterial and archaeal libraries used for further downstream analysis. Sequences stemming from ciliate 18S rRNA genes that were >200 bp in length were truncated to variable lengths so that the average quality score was >25 (all other parameters were set on the default option). The remaining sequences that did not meet the quality criteria were removed from the ciliate protozoal library.

Clustering of operational taxonomic units (OTUs) was performed using the uclust method [39] for bacteria and archaea at a 97% similarity threshold, or the prefix-suffix method passing the option “-p 1000” for protozoa (QIIME team, unpublished). Representative OTUs were assigned to taxonomic ranks as follows: bacterial 16S rRNA genes were BLAST-searched against the Greengenes database (gg_13_5/gg_13_5.fasta [38]); archaeal 16S rRNA genes and protozoal 18S rRNA genes were BLAST-searched against a rumen specific, in-house database [40] and the Silva eukaryotes database v.111 [41], respectively.

Of the samples collected in the study, 71/72 samples had at least 1000 bacterial sequences per sample. The remaining one sample had 268 sequences and was removed from the bacterial library used for further downstream data analysis. Within the archaeal library, 70/72 samples had at least 320 sequences per sample. The remaining two samples, which had 15 and 236 sequences, were removed from the archaeal library used for downstream data analysis. Since PCR amplicons for ciliate 18S rRNA genes were obtained from only 36/72 DNA samples, we used a minimum sequence read cut-off of 250 sequences per sample to report ciliate diversity in 26/36 samples. Subsequently, the OTU tables were rarefied at 1,000 (bacteria), 320 (archaea) and 250 (ciliate protozoa) sequences per sample and relative abundance tables were obtained at the phylum-, family- and genus-levels (bacteria), a mixed-taxon-level (archaea), or genus-level (ciliate protozoa).

To access the richness of microbial species captured within the samples, collector’s curves for bacteria, archaea and ciliate protozoa communities were constructed from the OTU tables generated in QIIME, by using the alpha_diversity.py script and the observed species metric. The alpha-diversity rarefaction analysis was computed for 1000 sequences per sample for bacteria which included 71/72 samples, 320 sequences for archaea (including 70/72 samples) and 250 sequences for ciliate protozoa (including 26/36 samples). The collector’s curves for the three microbial groups were visualised in SigmaPlot (2008 version 11, Systat Software, Inc., San Jose, CA, USA) by plotting the mean number + SD of OTUs observed against the number of sequences sampled.

When considering faecal samples from Groups A and B within each microbial group, bacterial phyla with relative abundances <1% in all samples were grouped as “Other Phyla”. Similarly, bacterial families or genera with relative abundances <1% in all samples were grouped as “Other Families” or “Other Genera”, respectively. The archaeal community was categorised at a mixed-taxon level, and archaeal clades with relative abundances <1% in all samples were grouped as “Other Taxa”. The ciliate protozoa community was categorised at genus level and all ciliate protozoa genera with relative abundances <1% in all samples were grouped as “Other Genera”. Sequence data generated in this study were deposited in the NCBI SRA under study accession number SRP033608.

### Statistical analyses

Alpha-diversity was evaluated at the OTU level using the QIIME pipeline [42]. The sampling completeness was evaluated by using the Good’s coverage estimator, which calculates the probability that a randomly selected amplicon sequence from a sample has already been sequenced [43], [44]. Good’s coverage [43] was calculated in Excel (version 2010, Microsoft Corp., Redmond, WA, USA), and presented as mean percentage ± SD for each microbial group. Additional diversity indices (species richness, species evenness, Shannon-Wiener’s diversity index, Simpson’s index of diversity) were calculated for the various levels (phylum, family and genus) for the bacterial community and a mixed-taxonomic level for the archaeal community, using the PAST software [45]. The species richness was evaluated by counting the number of taxa in the community and Pielou’s species evenness was calculated to explain the biodiversity in each sample by quantifying the species equality based on the distribution of relative abundances of the species in the community (ranging from 0–1, where 1 was complete evenness with least variation in the community). The Shannon-Wiener diversity index [46] was computed to explain the entropy, taking into account the species richness and evenness of the community, which varied from 0 for communities with a single taxon, to high values of ∼4.6 for highly diverse communities. Simpson’s index of diversity (1-D) [47] was used to describe the diversity in a community, ranging from 0–1, with 1 indicating maximum diversity in a sample.

Beta-diversity was evaluated on a genus level for the bacterial community and a mixed-taxonomic level for the archaeal community, using the QIIME pipeline. Only microbial taxa that represented ≥1% of the total community, in at least one sample within each microbial group (bacteria and archaea), were included in the downstream analysis. Differences in bacterial and archaeal communities between samples were calculated using the Bray-Curtis (which takes into account the presence or absence of a species and the relative abundance) and Sørensen-Dice (which takes into account the presence or absence of a species) dissimilarity metrics. Principal coordinate analysis (PCoA) was performed in QIIME and the clustering of samples based on the first two principal coordinates was visualised in SigmaPlot. Unweighted Pair Group Method with Arithmetic Mean (UPGMA) clustering was performed in QIIME, based on the Bray-Curtis dissimilarity matrix, to visualise the clustering of horses by diet on Days 0 and 4 of the study period, and the dendrograms were visualised in MEGA5 (version 5.2) [48].

The data generated in QIIME and PAST were imported into Excel, and re-formatted where necessary, before tests for statistical significance were conducted in STATA version 12.1 (Stata Corp, College Station, TX, USA). Non-normally distributed data are presented as median percentage and IQR throughout, and the non-parametric Kruskal-Wallis test was used to test for differences between the relative abundances of taxa identified in the faecal microbiomes of horses in Groups A and B. The level of significance used for differences between the bacterial and archaeal diversity indices on Days 0 and 4 was *P*<0.017 after Bonferroni adjustment for multiple comparisons. The relative abundances of bacterial communities between groups and within each group, on Days 0 and 4, were compared at multiple taxonomic levels (phylum, family and genus), with significance levels of *P*<0.004 (phylum level) and *P*<0.001 (family and genus levels) after Bonferroni adjustment for multiple comparisons. The relative abundances of the taxa in the archaeal communities between groups and within each group, on Days 0 and 4, were compared at a mixed-taxonomic level, with a significance level of *P*<0.008 after Bonferroni adjustment for multiple comparisons.

Inter-group (horses in Group A and B) and intra-group (horses in the same group) variation in bacterial and archaeal community structure on Day 0 were compared using the Bray-Curtis dissimilarity matrix to create a median value for each set of comparisons. The inter-horse (between horses in Group A and B on multiple sampling days) and intra-horse (horses compared with self on multiple sampling days) variation on Days 4–21 were also compared using Bray-Curtis dissimilarity to create a median value for each set of comparisons. The non-parametric Kruskal-Wallis test was used to test differences between the median values for each set of comparisons and the level of statistical significance was *P*<0.05.

Data for analysing the community structure of faecal microbiota (bacteria, archaea and ciliate protozoa) of pasture-fed horses are reported. The relative abundance data generated from QIIME were extracted, reformatted in excel to exclude the values for Day 0 in both Group A and B, for each microbial group, and the results presented as a median percentage and IQR. Diversity indices were calculated using the PAST software for the ciliate protozoa data for pasture-fed horses, after excluding the values for Day 0 in both Groups A and B, and presented as median and IQR.

## Results

Amongst the prokaryote domains, PCR amplicons of bacteria and archaea were obtained from all 72 faecal samples. Despite repeated testing of variable dilutions of DNA template concentrations, PCR amplicons of ciliate protozoa were only obtained from half of the faecal samples (36/72), which was insufficient to adequately represent the two groups of horses on each sampling day. The results of the phylogenetic analysis for the ciliate protozoa are therefore described as part of the faecal microbiota of pasture-fed horses, but are excluded from downstream analysis involving diet-specific comparisons.

### Metrics of pyrosequencing data for three microbial groups

The 72 faecal samples generated just under a million sequence reads (981,946) for the pooled microbial communities (bacteria, archaea and protozoa). Quality filtering and barcode mapping through the QIIME pipeline resulted in 553,715 sequences, the majority of which were, as expected from the pooling ratio, from the bacterial group (73%, 401,996), and the remaining sequences were archaea (71,403) and ciliate protozoa (80,316) (Table 1). Denoising of the bacterial and archaeal sequences resulted in 387,603 and 66,273 sequences respectively, and after removal of potential chimeras the number was further reduced to 387,083 and 65,639 sequences for bacteria and archaea respectively (Table 1). A total of 86,692 unique OTUs for bacteria and 63 unique OTUs for archaea were identified at 97% sequence similarity, from the total number of sequences obtained after chimera removal, and 36,896 unique OTUs were identified at 100% sequence similarity from all sequences for ciliate protozoa. The OTU tables for each microbial group were rarefied leaving a total number of 25,309 and 48 unique OTUs identified at 97% sequence similarity for bacteria and archaea, respectively, and 3,851 unique OTUs identified at 100% sequence similarity for the ciliate protozoa.

**Table 1:** Metrics of data generated by 454 GS FLX Titanium pyrosequencing of 16S and 18S rRNA gene amplicons from microbial groups present in 72 equine faecal samples.

Details	Microbial Group		
	Bacteria	Archaea	Ciliate protozoa
**Sequences after quality-filtering**			
Number of sequences	401,996	71,403	80,316
% of total sequences	72.6%	12.9%	14.5%
Mean number of sequences per sample	5,584	992	2171
(range)	(268–15,493)	(16–2,608)	(1–10,736)
Mean length of sequences	521.2	506.2	516.6
Mean length of sequences after removal of primers	470.8	452	483.6
Number of sequences after denoising	387,603	66,273	*
Number of sequences after chimera removal	387,083	65,639	*
**Sequences after sub-sampling** §			
Mean number of sequences per sample after sub-sampling	5,448	934	3,084
(range)	(1,333–15,006)	(322–2,438)	(252–10,736)
Number of sequences per sample (rarefied)	1000	320	250

*Not applicable (see Materials and Methods section).

§ Samples with low number of sequences were excluded from the microbial libraries (see Materials and Methods section).

Within the domain Bacteria, 19 phyla were detected, which encompassed at least 93 different families and 158 different genera (Table S2A). In Groups A and B, just over half of the phyla (10/19) had relative abundances ≥1% in at least one sample and the remaining phyla (9/19) had relative abundances <1% in all samples (collectively referred to as “Other Phyla”). Two thirds of the families (65/93) had relative abundances <1% (“Other Families”), leaving a third of the families (28/93) with relative abundances ≥1% in at least one sample. The majority of genera (118/158) had relative abundances <1% (“Other Genera”), the remaining genera (40/158) had relative abundances ≥1% in at least on sample. Several organisms were detected, which are as yet “unclassified” in the Greengenes database. In the domain Archaea, two phyla were detected, encompassing at least five different families and 10 different clades (Table S2B). Ciliate protozoa were detected in 26 out of 72 samples and belonged to at least 15 different genera (Table S2C).

### Rarefaction analysis and coverage of microbial diversity

**Figure 1:**
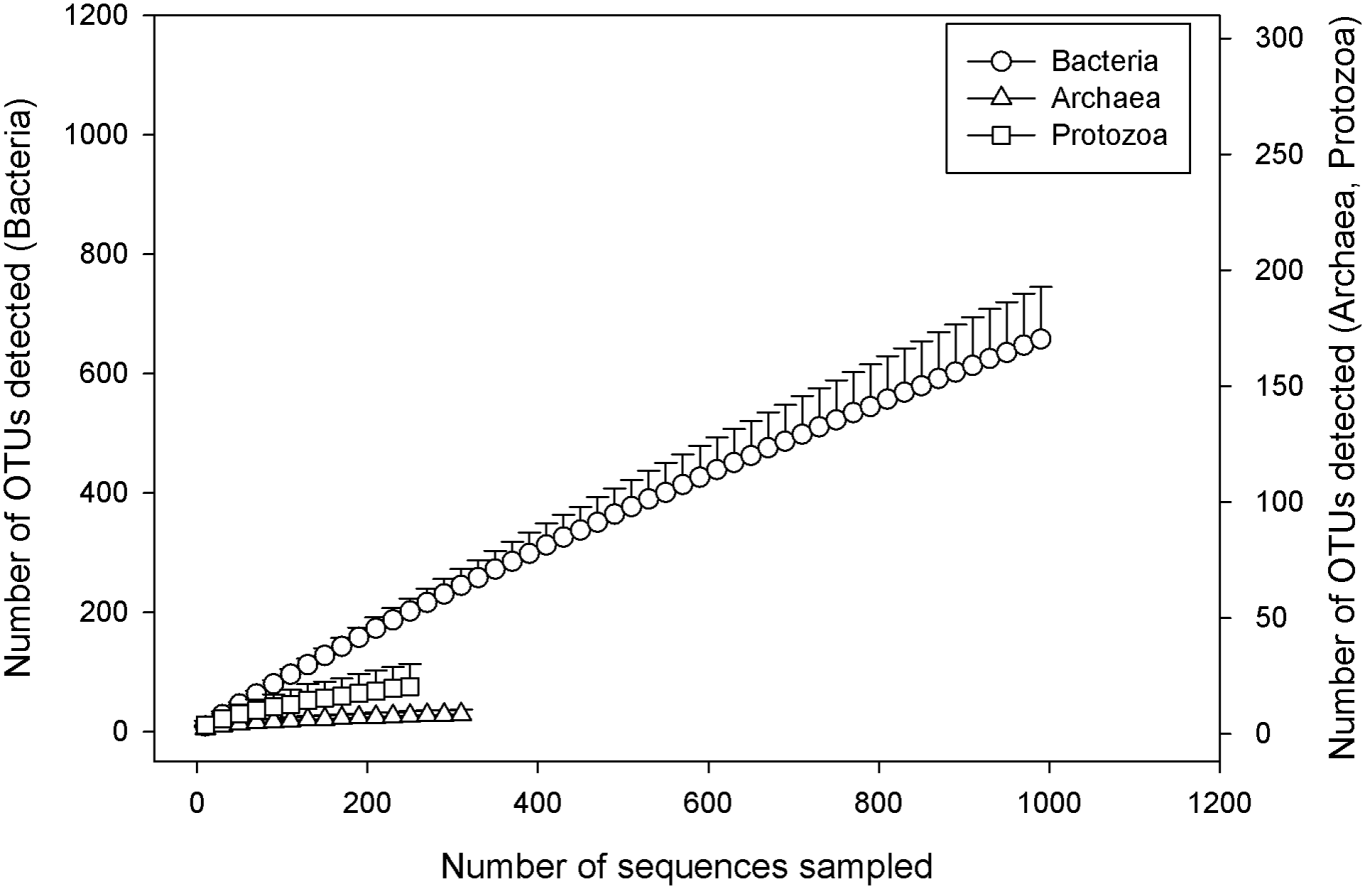
Rarefaction curves for microbial communities in faecal samples of forage-fed horses. The rarefaction curves show the mean number (with standard deviation) of observed species against the depth of sequencing of bacterial (-ο-), archaeal (-Δ-) and ciliate protozoal (--) communities in the equine faeces sampled in the study (n = 72 faecal samples). Multiple rarefactions were calculated from the OTU tables obtained for each of the three microbial groups representing 71 out of 72 samples for bacteria (minimum of 1,000 sequence reads/sample), 70 out of 72 samples for archaea (minimum of 320 sequence reads/sample) and 26 out of 37 samples for ciliate protozoa (minimum of 250 sequence reads/sample).

Rarefaction analysis for the three microbial groups is presented in Figure 1. There was a plateau in the number of new OTUs detected when a minimum of approximately 320 sequences per sample and 250 sequences per sample were rarefied for the archaeal and ciliate protozoal groups, respectively. The collector’s curve for the bacterial group had still not reached an asymptote when rarefied at a minimum of approximately 1,000 sequences per sample (Figure 1, S1A, B). However, Good’s coverage estimates indicated that the sampling depth had adequately captured a large part of the species diversity in all three microbial groups, with the mean coverage being 99.60±0.17% for the bacterial community, 99.94±0.07% for the archaeal community, and 99.90±0.11% for the ciliate protozoal community.

### Composition of the faecal bacterial community pre-dietary change

#### Diversity Indices

There was a difference in the median Simpson’s indices of diversity (1-D) of bacterial genera in Group A (0.80 [IQR 0.79–0.82]) and Group B (0.85 [IQR 0.84–0.85]) (*P* = 0.016). There were no significant differences between Group A and B for the median Shannon-Wiener (2.22 [IQR 2.07–2.33] and 2.35 [IQR 2.28–2.38]; *P* = 0.149) and evenness (0.31 [IQR 0.28–0.31] and 0.38 [0.31–0.32]; *P* = 0.423) diversity indices.

#### Comparison of relative abundances of taxa in the bacterial community at multiple levels

**Figure 2:**
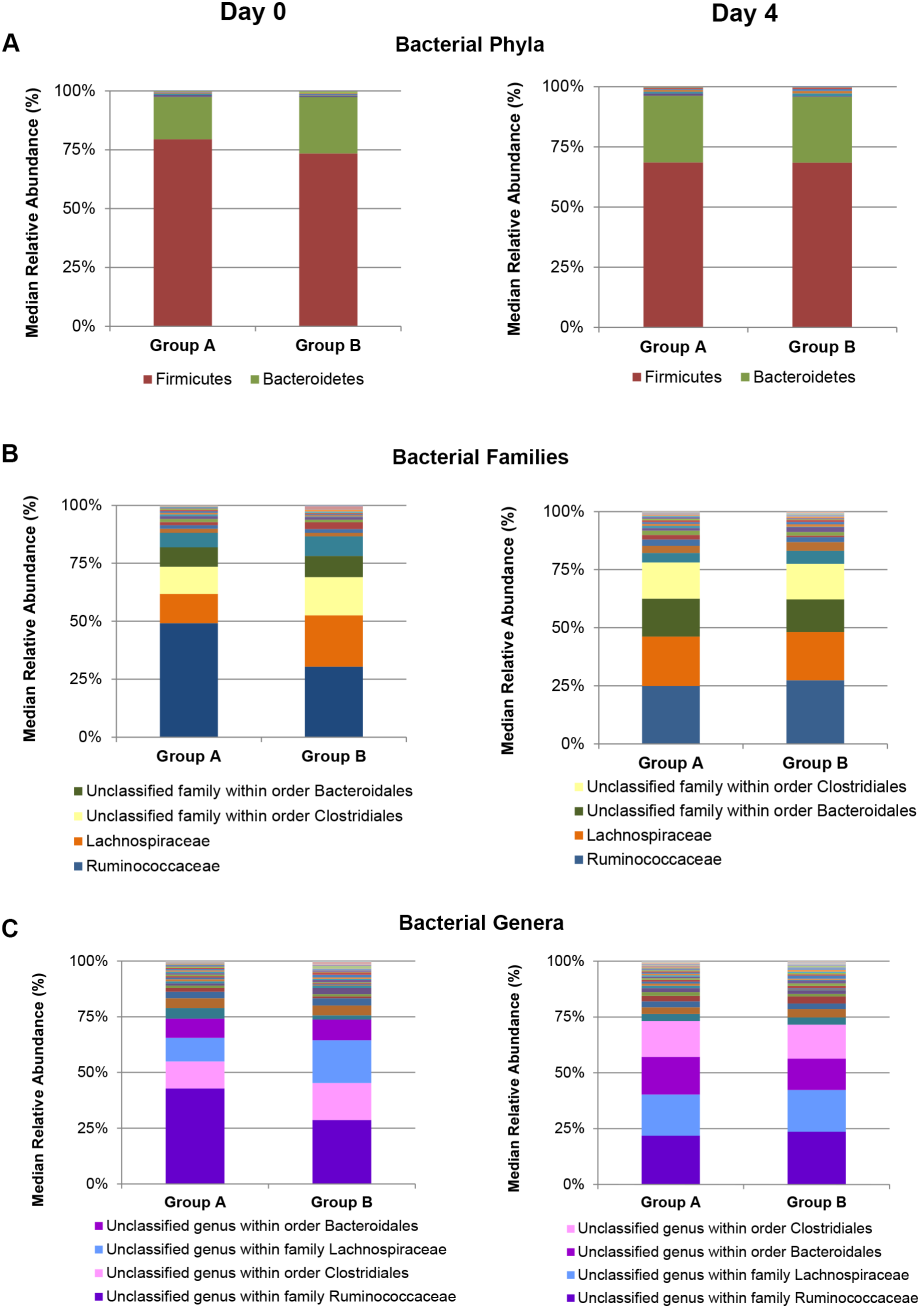
Comparison of the bacterial community structure at multiple levels. The median relative abundances of the bacterial phyla in the faeces of Group A and B horses, on Days 0 and 4, are illustrated in the figure, panel (A). Panel (B) shows the relative abundances of the bacterial families, and panel (C) shows the relative abundances of bacterial genera present in the faeces of Group A and B horses, on Days 0 and 4. The stacked bar graph is presented in ascending order of the median relative abundances of bacterial taxa for Group A horses, and the legends show the most dominant taxa in each graph (>15% median relative abundance for the phylum level and >8% for the family and genus levels).

Two phyla, namely the Firmicutes and Bacteroidetes dominated the bacterial community, with median relative abundances of 80% (IQR 67–84) and 18% (IQR 13–27) in Group A horses and 74% (IQR 70–74) and 24% (22–28) in Group B horses, respectively (Figure 2A). There were no significant differences detected among bacterial phyla between the two diet-groups (Table 2). Members of the family Ruminococcaceae had the highest median relative abundance in both Group A (47% [IQR 43–53]) and Group B (30% [IQR 25–33]), followed by members of the family Lachnospiraceae (12% [IQR 8–18] in Group A; 22% [IQR 18–26] in Group B) and members of as yet unclassified families within the orders Clostridiales (11% [IQR 10–13]; 16% [IQR 15–18]) and Bacteroidales (8% [5]–[10]; 9% [8]–[13]), each in Groups A and B, respectively (Figure 2B). For the as yet unclassified members within the order Clostridiales, there was a difference in the median relative abundances between Groups A and B (*P* = 0.003).

**Table 2:** Comparison of the relative abundances of bacterial phyla in the faeces of Group A (fed Diet F, n = 6) and Group B (fed Diet P, n = 6) horses on Day 0, and Day 4 (both groups fed Diet P).

	Relative abundance on Day 0					Relative abundance on Day 4				
	Group A		Group B			Group A		Group B		
Bacterial Phylum	Median	IQR^a^	Median	IQR^a^	*P*-value^b^	Median	IQR^a^	Median	IQR^a^	*P-*value^b^
Firmicutes	0.797	0.669–0.838	0.736	0.704–0.740	0.420	0.684	0.631–0.750	0.680	0.621–0.728	1.000
Bacteroidetes	0.181	0.131–0.273	0.240	0.216–0.278	0.260	0.276	0.222–0.298	0.272	0.223–0.335	0.870
Spirochaetes	0.006	0.004–0.007	0.003	0.002–0.004	0.140	0.004	0.002–0.005	0.008	0.004–0.010	0.060
Cyanobacteria	0.004	0.001–0.004	0.001	0.000–0.002	0.120	0.002	0.000–0.002	0.002	0.001–0.007	0.420
Fibrobacteres	0.003	0.002–0.005	0.005	0.003–0.009	0.200	0.008	0.004–0.010	0.010	0.008–0.017	0.200
Proteobacteria	0.003	0.002–0.004	0.003	0.001–0.004	0.800	0.003	0.002–0.005	0.002	0.001–0.003	0.170
Actinobacteria	0.002	0.001–0.004	0.009	0.007–0.021	0.010	0.008	0.006–0.009	0.012	0.010–0.012	0.020
Armatimonadetes	0.001	0.000–0.001	0.002	0.001–0.003	0.130	0.009	0.006–0.017	0.002	0.001–0.002	0.010
Planctomycetes	0.001	0.000–0.003	0.000	0.000–0.000	0.180	0.001	0.000–0.002	0.001	0.000–0.001	0.470
Synergistetes	0.000	0.000–0.002	0.000	0.000–0.001	0.700	0.001	0.000–0.001	0.000	0.000–0.001	0.630
Other Taxa	0.008	0.005–0.008	0.005	0.004–0.005	0.030	0.004	0.003–0.005	0.006	0.003–0.006	0.470

The bacterial phyla are listed in descending order of relative abundances for Group A horses on Day 0, and all phyla present at relative abundances of <1% in all samples are grouped as Other Taxa.

a IQR–Interquartile range.

b Level of statistical significance after Bonferroni adjustment for multiple comparisons *P* = 0.004.

At genus level, the highest median relative abundances were of as yet unclassified members within the family Ruminococcaceae (40% [IQR 39–41; 28% [IQR 23–29]), unclassified members within the order Clostridiales (11% [IQR 10–13]; 16% [IQR 15–18]), unclassified members within the family Lachnospiraceae (10% [IQR 7–14]; 19% [IQR 16–24]), and unclassified members within the order Bacteroidales (8% [IQR 5–10]; 9% [IQR 8–13]), each in Groups A and B, respectively (Table 3; Figure 2C). There was a difference between Groups A and B in the relative abundances of as yet unclassified members within the order Clostridiales (*P* = 0.003) and within the family Lachnospiraceae (*P* = 0.006), and the less abundant genera CF231 (*P* = 0.004) and BF311 (*P* = 0.003) (Table 3, S3A).

**Table 3:** Comparison of the relative abundances of bacterial genera in the faeces of Group A (fed Diet F, n = 6) and Group B (fed Diet P, n = 6) horses on Day 0.

	Relative abundance on Day 0				
Taxonomic rank within the domain Bacteria	Group A		Group B		
Phylum > Class > Order > Family > Genus	Median	IQR^a^	Median	IQR^a^	*P-*Value^b^
Firmicutes > Clostridia > Clostridiales > Ruminococcaceae > unclassified	0.395	0.387–0.413	0.281	0.227–0.293	0.016
Firmicutes > Clostridia > Clostridiales > unclassified > unclassified	0.112	0.103–0.131	0.163	0.149–0.181	0.003*
Firmicutes > Clostridia > Clostridiales > Lachnospiraceae > unclassified	0.098	0.067–0.139	0.188	0.157–0.238	0.006*
Bacteroidetes > Bacteroidia > Bacteroidales > unclassified > unclassified	0.079	0.053–0.100	0.091	0.080–0.132	0.688
Bacteroidetes > Bacteroidia > Bacteroidales > [Paraprevotellaceae] > YRC22	0.040	0.006–0.063	0.043	0.037–0.067	0.470
Firmicutes > Clostridia > Clostridiales > Ruminococcaceae > *Ruminococcus*	0.045	0.028–0.058	0.019	0.015–0.030	0.054
Bacteroidetes > Bacteroidia > Bacteroidales > [Paraprevotellaceae] > CF231	0.002	0.000–0.003	0.012	0.008–0.022	0.004*
Bacteroidetes > Bacteroidia > Bacteroidales > Bacteroidaceae > BF311	0.001	0.000–0.001	0.010	0.009–0.014	0.003*

The table lists bacterial genera that were present at relative abundances of ≥4% in both Groups A and B, and certain genera (present at <4% relative abundance) that were different between Groups A and B on Day 0. The taxonomic ranks are listed from Phylum to Genus in descending order of relative abundances for Group A.

a IQR–Interquartile range.

b Level of statistical significance after Bonferroni adjustment for multiple comparisons *P* = 0.001.

*Differences between Groups A and B.

#### Beta diversity

**Figure 3:**
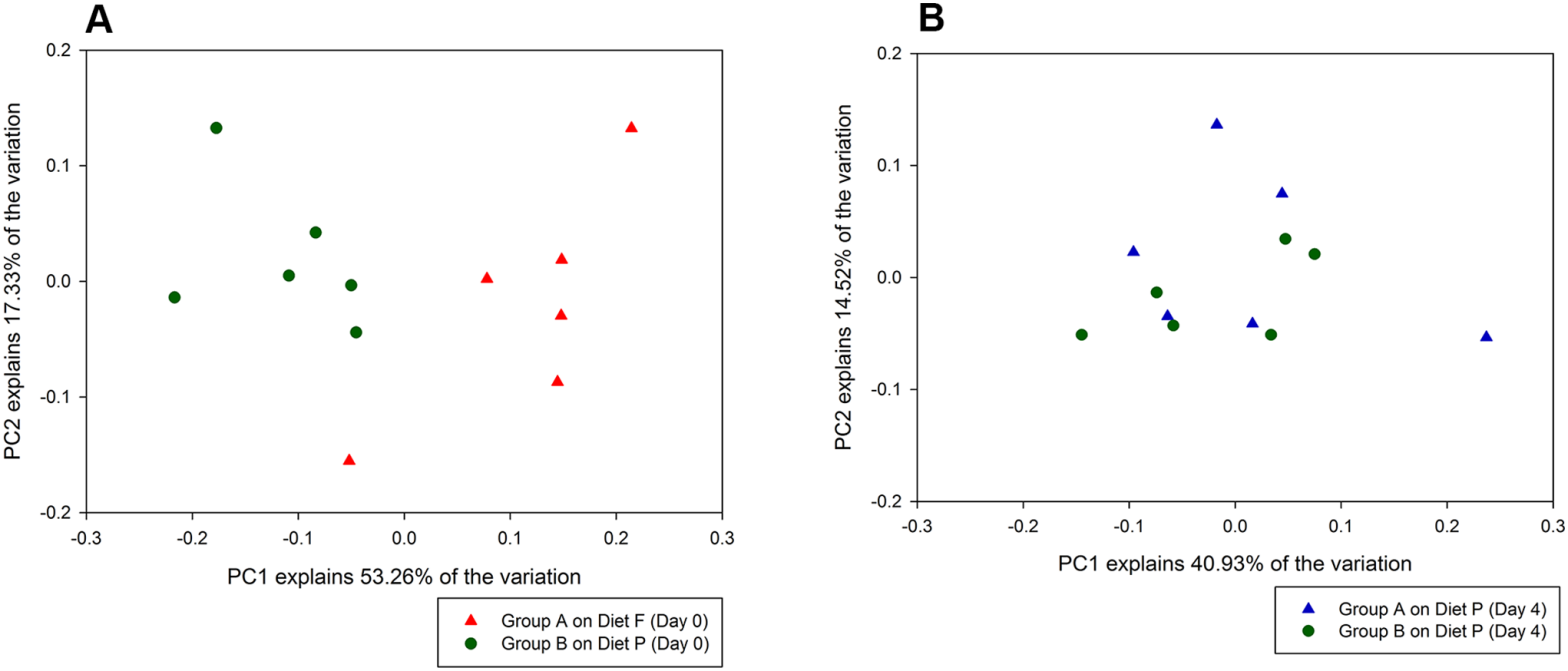
Principal coordinate analysis (PCoA) of bacterial communities detected in equine faeces pre- and post-dietary change based on the Bray-Curtis dissimilarity metric. (A) PCoA plot of faecal bacterial communities for Group A (Diet F) and Group B (Diet P) horses illustrates clustering of horses by dietary treatment group at Day 0. (B) PCoA plot of faecal bacterial communities for Group A (transition from diet F to diet P) and Group B (Diet P) horses illustrates no clustering by dietary treatment group at four days post-dietary change. Explanations for the symbols used are given in the legend.

**Figure 4:**
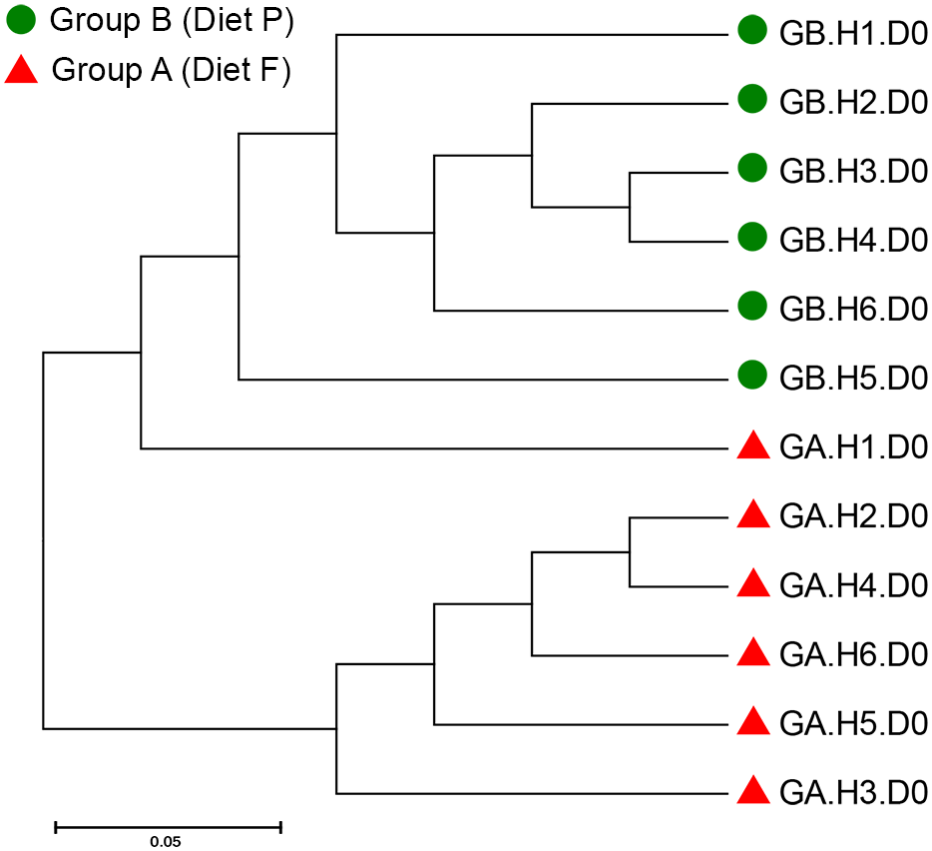
UPGMA dendrogram based on the Bray-Curtis dissimilarity matrix on Day 0. The Bray-Curtis dissimilarity metric takes into account the presence and absence of taxa and the relative abundances of the taxa, to generate a dissimilarity matrix for the faecal microbiota present in samples from horses on Day 0. The UPGMA dendrogram shows distinct clustering of horses (H; numbered from 1–6 per group) by group, indicating that the faecal bacterial community structure of horses on Day 0 differed between Group A (GA) and Group B (GB), which were fed Diet F and P respectively.

Principal coordinate analysis on genus level using Bray-Curtis dissimilarity (which takes into account presence and absence as well as relative abundance of a taxonomic group) revealed clustering of horses by dietary treatment group with more than half (53.3%) of the variation explained by PC1 and 17.3% of the variation explained by PC2 (Figure 3A). Similarly, UPGMA dendrograms showed distinct clusters of horses by diet (Figure 4). The median inter-group dissimilarity of faecal bacterial communities (0.298 [IQR 0.235–0.347]) was significantly higher (*P* = 0.0004) than the median intra-group dissimilarity (0.220 [IQR 0.180–0.265]) of horses on Day 0. Clustering by diet was not observed in UPGMA dendrograms using the Sørenson-Dice dissimilarity metric, which only takes into account presence and absence of a taxonomic group (data not shown).

### Dynamic adaptation of the faecal bacterial community post-dietary change

#### Diversity Indices

At genus level, the median Simpson’s indices of diversity (1-D) were similar between bacterial communities in Groups A (0.87 [IQR 0.86–0.89]) and B (0.86 [IQR 0.85–0.87]) on Day 4 (*P* = 0.425). There were no significant differences in the median Shannon-Wiener (2.50 [IQR 2.43–2.56]; 2.43 [IQR 2.42–2.49]; *P* = 0.335) and the evenness (0.35 [IQR 0.32–0.38]; 0.33 [IQR 0.32–0.36]; *P* = 0.262) diversity indices between Groups A and B, respectively. There were significant differences in the median Simpson’s indices of diversity of Group A horses between Day 0 (0.80 [IQR 0.79–0.82]) and Day 4 (0.87 [IQR 0.86–0.89]); (*P* = 0.004). The median Shannon-Wiener diversity indices of Group A horses on Day 0 (2.22 [IQR 2.07–2.33) and Day 4 (2.47 [IQR 2.43–2.56]) were significantly different (*P = *0.004). There were no differences in the evenness diversity indices of Group A horses between Days 0 and 4. There were no significant differences in the diversity indices of Group B horses on Days 0 and 4 (data not shown).

#### Comparison of relative abundances of taxa in the bacterial community at multiple levels

The bacterial community was dominated by two phyla, Firmicutes and Bacteroidetes, with median relative abundances of 68% (IQR 63–75) and 28% (IQR 22–30) in Group A horses and 68% (IQR 62–73) and 27% (IQR 22–34) in Group B horses, respectively (Figure 2A). There were no significant differences between the relative abundances of bacterial phyla detected in the faeces of Group A and B horses on Day 4 (Table 2). Members of the family Ruminococcaceae had the highest relative abundance in both Group A (24% [IQR 20–27]) and Group B (26% [IQR 22–33]), followed by members of the family Lachnospiraceae (21% [IQR 17–25] in Group A; 20% [IQR 19–22] in Group B), members of as yet unclassified families within the orders Bacteroidales (16% [IQR 13–18]; 13% [IQR 12–18]) and Clostridiales (15% [IQR 13–17]; 15% [IQR 13–16]), each in Groups A and B, respectively (Figure 2B). There were no significant differences between the relative abundances of bacterial families detected in the faeces of Groups A and B horses on Day 4 (data not shown).

At genus level, the highest relative abundances in Groups A and B were of as yet unclassified members within the family Ruminococcaceae, unclassified members within the family Lachnospiraceae, and unclassified members within the orders Bacteroidales and Clostridiales (Figure 2C, Table S3A). Only one genus (RFN20, belonging to the phylum Firmicutes, family Erysipelotrichaceae), present at a low abundance (<2%), showed a difference in relative abundance between Groups A and B on Day 4 (*P* = 0.004; Table S3A). When comparing the faecal bacterial community of Group A horses, the relative abundances of three bacterial genera (*Pseudoramibacter Eubacterium* and as yet unclassified members within the family Ruminococcaceae and within the order RB046 of the phylum Armatimonadetes) differed between Day 0 and Day 4 (Table 4, S3B). There were no significant differences between the bacterial genera detected in the faeces of Group B horses that were grazing pasture on both Days 0 and 4 (Table S3C).

**Table 4:** Comparison of the relative abundances of bacterial genera in the faeces of Group A horses on Day 0 (fed Diet F, n = 6) and Day 4 (fed Diet P, n = 6).

Taxonomic rank within the domain Bacteria	Relative abundance of Group A horses				
Phylum > Class > Order > Family > Genus	Day 0		Day 4		
	Median	IQR^a^	Median	IQR^a^	*P-*Value^b^
Firmicutes > Clostridia > Clostridiales > Ruminococcaceae > unclassified	0.395	0.387–0.413	0.205	0.171–0.220	0.004*
Firmicutes > Clostridia > Clostridiales > unclassified > unclassified	0.112	0.103–0.131	0.151	0.130–0.167	0.078
Firmicutes > Clostridia > Clostridiales > Lachnospiraceae > unclassified	0.098	0.067–0.139	0.173	0.151–0.195	0.025
Bacteroidetes > Bacteroidia > Bacteroidales > unclassified > unclassified	0.079	0.053–0.100	0.158	0.132–0.177	0.109
Firmicutes > Clostridia > Clostridiales > Ruminococcaceae > *Ruminococcus*	0.045	0.028–0.058	0.025	0.018–0.032	0.146
Bacteroidetes > Bacteroidia > Bacteroidales > [Paraprevotellaceae] > YRC22	0.040	0.006–0.063	0.023	0.017–0.025	0.749
Firmicutes > Clostridia > Clostridiales > Eubacteriaceae > *Pseudoramibacter_Eubacterium*	0.001	0.000–0.002	0.006	0.004–0.007	0.004*
Armatimonadetes > SJA-176> RB046> unclassified > unclassified	0.001	0.000–0.001	0.009	0.006–0.017	0.005*

The table lists bacterial genera that were present at relative abundances of ≥4% in the faeces of Group A horses, and certain genera (present at <4% relative abundance) that were different between Days 0 and 4. The taxonomic ranks are listed from Phylum to Genus in descending order of relative abundances for Day 0.

a IQR–Interquartile range.

b Level of statistical significance after Bonferroni adjustment for multiple comparisons *P* = 0.001.

*Differences between Days 0 and 4.

#### Beta diversity

**Figure 5:**
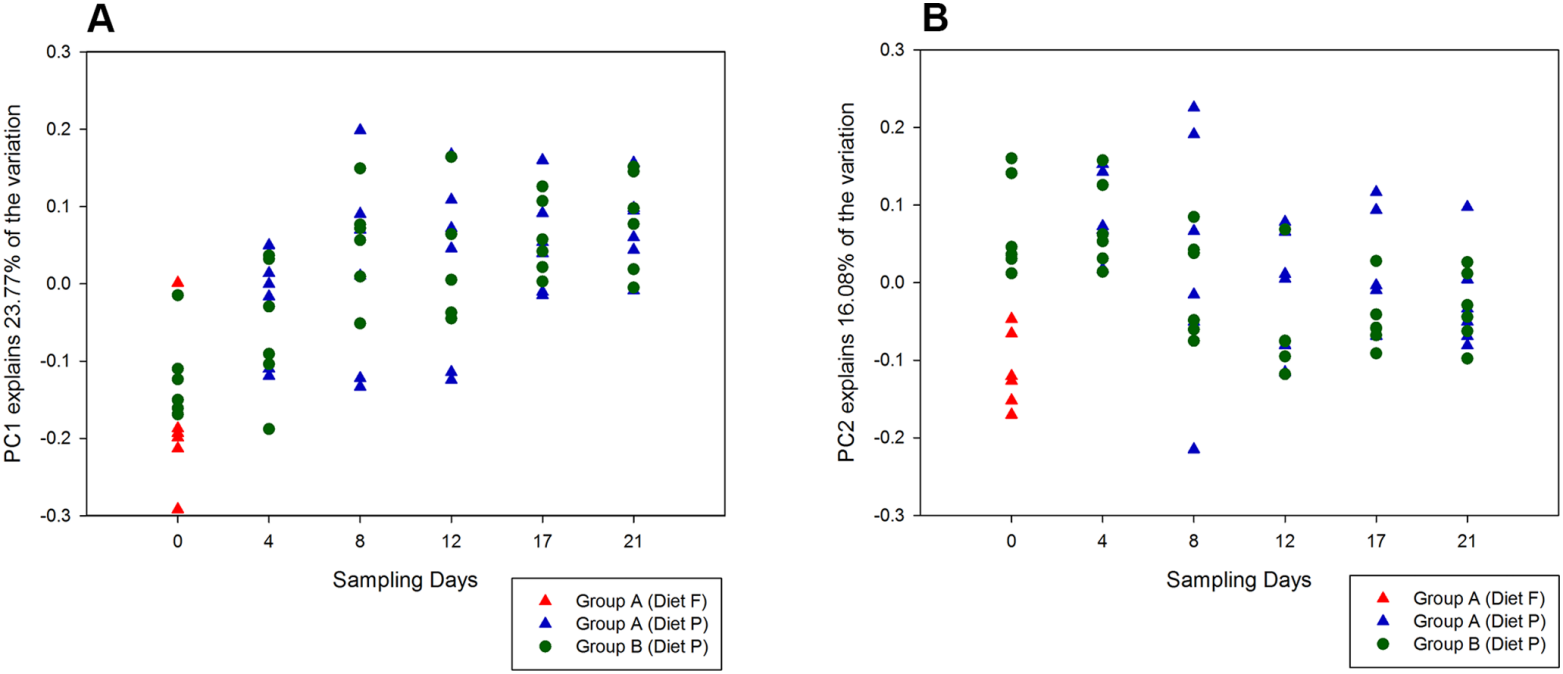
Principal coordinate analysis (PCoA) of bacterial communities detected in equine faeces over 3-weeks based on the Bray-Curtis dissimilarity metric. The plots illustrate the structure of the faecal bacterial communities in horses of Groups A and B on six sampling time-points over a period of three weeks. The plots show group-wise clustering of horses fed Diet F and Diet P on Day 0, and no clustering when horses were fed pasture (Diet P) from Days 4–21, with 24% variation explained on PC1 (A) and 16% variation explained on PC2 (B).

On Day 4, bacterial communities did not cluster by diet-group (Figure 3B). Furthermore, there was no clustering by group from Day 4 through to Day 21 (Figure 5). Diet-specific clusters were observed between the horses fed Diet F (Group A horses on Day 0) and those fed pasture (Groups A horses on Days 4–21 and Group B horses on Days 0–21), (Figure S4). Inter- and intra-horse variation across all horses on pasture (Days 4–21 of both groups) had a median dissimilarity of 0.236 (IQR 0.189–0.291) and 0.222 (IQR 0.176–0.276), respectively (*P* = 0.044).

### Composition of the faecal archaeal community pre- and post-dietary change

The median Simpson’s indices of diversity were similar for both groups A (0.48 [IQR 0.19–0.55]; 0.42 [IQR 0.40–0.48]) and B (0.42 [IQR 0.36–0.46]; 0.45 [IQR 0.41–0.49]), on Days 0 and 4 respectively. The median Shannon-Wiener diversity indices for Group A (0.81 [IQR 0.44–0.95]; 0.70 [IQR 0.67–0.70]) and Group B (0.68 [IQR 0.60–0.72]; 0.70 [IQR 0.68–0.75]), and the evenness diversity indices for Group A (0.5 [IQR 0.31–0.61]; 0.59 [IQR 0.44–0.69]) and Group B (0.51 [IQR 0.45–0.68]; 0.54 [IQR 0.49–0.68]) on Days 0 and 4 respectively, were also similar. There were no significant differences in the diversity indices of the faecal archaeal community between Group A horses on Days 0 and 4, between Group B horses on Days 0 and 4, and between Group A and B horses on either Day 0 or Day 4 (data not shown).

Over half (6/10) of the archaeal clades in Groups A and B were present at a relative abundance of ≥1% in at least one sample, and the remaining clades were grouped as “Other Taxa”. The archaeal community was dominated by two clades, *Methanocorpusculum* and relatives and *Methanobrevibacter ruminantium* and relatives in both groups, on both Day 0 and Day 4 (Figure S2, Table S4A, B). There were no significant differences in the median relative abundances of archaeal taxa between Group A horses on Days 0 and 4 (Table S4A), and Group B horses on Days 0 and 4 (Table S4B). There were no significant differences in the median relative abundances of archaeal taxa between Groups A and B on Day 0 or Day 4 (Table 5, S4C). Principal coordinate analysis did not show group-wise clustering of horses on Day 0 or on Days 4–21 (Figure S3). There was no significant difference in the inter-group or intra-group dissimilarity on Day 0 or on Days 4–21 (*P = *0.987).

**Table 5:** Comparison of the relative abundances of archaeal taxa (clade-level) in the faeces of Group A (fed Diet F, n = 6) and Group B (fed Diet P, n = 6) horses on Day 0.

Taxonomic rank under the domain Archaea	Relative abundance on Day 0				
Phylum > Class > Order > Genus/Clade	Group A		Group B		
	Median	IQR^a^	Median	IQR^a^	*P–*Value^b^
Euryarchaeota > Methanobacteria > Methanobacteriales > Methanobacteriaceae > *Methanobrevibacter* > *Methanobrevibacter_ruminantium*_and_relatives	0.630	0.413–0.897	0.663	0.569–0.769	0.710
Euryarchaeota > Methanomicrobia > Methanomicrobiales > *Genera_incertae_sedis* > *Methanocorpusculum*_and_relatives	0.177	0.056–0.309	0.319	0.219–0.431	0.200
Euryarchaeota > Thermoplasmata > Thermoplasmatales > Rumen Cluster C_and_relatives	0.016	0.003–0.031	0.016	0.009–0.038	0.850
Euryarchaeota > Methanobacteria > Methanobacteriales > Methanobacteriaceae > *Methanobrevibacter* > *Methanobrevibacter_gottschalkii*_and_relatives	0.014	0.000–0.028	0.000	0.000–0.003	0.120
Euryarchaeota > Methanobacteria > Methanobacteriales > Methanobacteriaceae > *Methanosphaera*	0.011	0.003–0.025	0.000	0.000–0.000	0.030
Other Taxa	0.000	0.000–0.000	0.000	0.000–0.000	0.360

a IQR–Interquartile range.

b Level of statistical significance after Bonferroni adjustment for multiple comparisons *P* = 0.008.

### Composition of the faecal microbiota of horses maintained exclusively on pasture

The faecal microbiota of all horses (Groups A and B) grazing on pasture from Days 4–21 comprised of a diversity of bacteria, archaea and ciliate protozoa. The highest median relative abundances of bacterial taxa were of unclassified members within the family Ruminococcaceae (22% [IQR 17–27]), the order Bacteroidales (21% [IQR 17–25]), the order Clostridiales (12% [IQR 10–14]) and within the family Lachnospiraceae (11% [IQR 10–15]), which together contributed two-thirds (66%) of the bacterial community. The bacterial genera detected at >1% relative abundance were *Bacteroides*, *Blautia*, *BF311*, *CF231*, *Clostridium*, *Coprococcus*, *Fibrabacter*, *Mogibacterium*, *Oscillospira*, *Paludibacter*, *Parabacteroides*, *Prevotella*, *Pseudoramibacter eubacterium*, *p-75-a5*, *RFN20*, *Ruminococcus*, *Treponema and YRC22*. The microbiota of pasture-fed horses included 10 archaeal genera and their clades, six of which were present at ≥1% in at least one sample. The remaining four genera and clades (including *Methanobacterium*, *Sulfolobus* and relatives, *Methanobrevibacter arboriphilus* and relatives and *Methanosarcina* and relatives) were present at <1% relative abundance (collectively referred to as “Other Taxa”). The highest median relative abundances were of two clades, *Methanocorpusculum* and relatives (77% [IQR 58–90]) and *Methanobrevibacter ruminantium* and relatives (17% [IQR 8–39]), which together contributed 94% of the archaeal community in the faeces of pasture-fed horses. The remaining 6% was of the clade Rumen Cluster C and relatives (median relative abundance 3% [IQR 2–4]) and the genera *Methanosphera*, *Methanimicrococcus* and Other Taxa, each at <1% median relative abundances.

Within the ciliate protozoa community (Table S2C), 15 genera under the class Litostomatea were identified as part of the faecal microbiota of pasture-fed horses. These included nine genera under the sub-class Trichostomatia present at ≥1% in at least one sample, and at least six other genera under the sub-classes Trichostomatia and Haptoria that were present at <1% relative abundance (collectively referred to as “Other Ciliate Genera”). The median Simpson’s index of diversity (1-D) for the ciliate community of pasture-fed horses was 0.66 (IQR 0.48–0.73), and the diversity indices for Shannon-Wiener and evenness were 1.4 (IQR 0.9–1.5) and 0.49 (IQR 0.40–0.61), respectively. The highest median relative abundance was represented by the genus *Spirodinium* (24% [IQR 7–41]), followed by the genera *Triadinium* (9% [IQR 4–22]) and *Cochliatoxum* (6% [IQR 1–11]). The other genera identified were *Blepharocorys*, *Bundleia*, *Cycloposthium* (each at ∼4% median relative abundance) and *Isotricha*, *Polydiniella*, and the group of Other Ciliate Genera (including *Didinium*, *Epiphyllum*, *Pelagodileptus*, *Diplodinium*, *Entodinium* and *Eremoplastron*) each at <1% median relative abundance.

## Discussion

The present study investigated changes in the faecal microbiota via pyrosequencing of 16S rRNA gene amplicons derived from microbes present in the faeces of two groups of yearling horses. The results demonstrated dietary effects on the bacterial communities in the faeces, with changes occurring in their relative abundances within four days following dietary transition.

In the present study, group-wise clustering of bacterial community structures on Day 0 was observed only by using the Bray-Curtis dissimilarity metric (Figure 3A and 4) and not the Sørensen-Dice dissimilarity metric, indicating that the differences in community structure observed in the PCoA plots were due to differences in the relative abundances of the bacterial genera, and not the presence or absence of certain genera in the faecal samples from horses fed either of the two diets. Due to PCR bias [49], there are some limitations in the use of sequencing techniques to compare the true diversity of microbial communities, as there is a possibility that true abundances in an ecosystem may differ from those detected. However, based on current literature, it appears fair to compare relative abundances of microbial populations in samples that have been processed equally. Therefore, it is reasonable to conclude that the initial differences observed between the faecal microbiota of horses in the two groups of the present experiment were linked to nutritional/dietary factors, given the convergence of the microbial community structure once on similar pasture diets.

The median Simpson’s index of diversity for the bacterial community in the faeces of horses fed the ensiled conserved forage-grain diet differed significantly from that of horses on pasture, and upon dietary transition, the diversity increased rapidly from 0.80 to 0.87 within four days of feeding on pasture. A high diversity in the microbiota may be a natural evolutionary strategy for survival of a cursorial herbivorous browser, providing the opportunity to rapidly respond to varying quantities and types of forage available at different time-points. Annual and seasonal variation has been shown to occur in the growth and composition of the rye-clover mix pasture, and the water-soluble carbohydrate content in the grass may change diurnally [50]. The moderate fluctuation in the bacterial community structure observed in the horses maintained on pasture in the present study may be a result of changes in the composition of pasture and warrants further investigation (Figure 5).

The transition, from different bacterial community structures at the start of the experiment to similar community structures when both groups were maintained on pasture, occurred rapidly within a 4-day period. This was a shorter transition time than the six days post-dietary change in faecal samples previously reported [23], and may be due to the use of a more sensitive technique in the present study. The dietary challenge in the present trial was moderate and primarily forage-based. The rapid change observed in the structure of the faecal bacterial community when horses were transitioned from feeding on an ensiled conserved forage-grain diet to pasture, and the subtle changes in the bacterial community structure observed when on pasture, emphasizes the sensitivity of the microbiota to changes in dietary substrate. Given the results of the present study, it may be suggested that the basal microbiota that inhabits the horse’s gastrointestinal tract is highly diverse and primed to respond to acute changes in diet. Hence, a shorter more intense sampling period around the time of dietary transition should be considered for future studies on the faecal microbiota of horses.

Bacteria are reported to form the largest proportion of the equine hindgut and faecal microbiomes [16] with the bacterial community dominated by the phyla Firmicutes and Bacteroidetes [13], [15], which was also observed in the present study. At Day 0, there were no differences between Group A and Group B in the beta-diversity of the bacterial communities at the phylum level; however, there were differences at higher taxonomic resolution (at family and genus levels) (Figure 2). This finding of differences in the beta-diversity at family and genus levels is in contrast to the findings of other studies where a mixed population of horses were fed a variety of diets [13], [14], [22], and may be due to the higher resolution possible with the use of the next generation sequencing technique.

The number of unclassified bacterial genera (68) detected in the present study demonstrates the paucity of knowledge on the composition of the microbiota (Table S2A). Therefore, further cultivation and non-cultivation based studies in various populations of horses are required to evaluate the abundance and occurrence of the as yet unclassified organisms and to understand their functional role in hindgut microbial fermentation.

Lactic acid bacteria of the genera *Lactobacillus* and *Streptococcus* are potentially associated with grain diets and have been widely reported within the literature [8], [51]. These genera have been implicated in the development of gastrointestinal disturbances and laminitis [13], [19], [20]. In the present study, the genus *Lactobacillus* was present at <1% relative abundance in the faecal microbiomes, whereas the genus *Streptococcus* was not detected in either group of horses, not even at relative abundances of <1%. Although a higher abundance of these genera may be detected in proximal regions of the hindgut than in the faeces, our findings are in contrast to many other reports in the literature (analysing caecal, colonic and faecal samples) when horses were fed grain: forage combinations or different types of forages [9], [21], [22], [23]. None of the horses in the present study showed signs of colic, laminitis, or digestive disturbances throughout the study period, or the three weeks prior to Day 0. Given that grain diets are associated with shifts in microbial populations [52], the low abundance or non-detection of the lactic acid bacteria in the present study could be related to the dietary history of the yearling horses, wherein the management was primarily pasture-based and large quantities of grain had not been offered. There are indications that providing good quality pasture and increased activity may play a role in buffering the negative effects of grain supplementation on the hindgut microbiota [53]. It is not clear what quantity of grain or soluble carbohydrate content in pasture results in shifts in hindgut microbial populations, and this requires further investigation.

The present study targeted multiple domains of microorganisms and provided new information about the influence of diet on the structure of the Bacteria and other members of the eHGM, such as the archaea and ciliate protozoa. The diversity of the archaeal community was limited in the present study (Figure 1), and similar to previous studies [32], [52], with sequence reads being assigned to only 10 different archaeal clades. The presence of the two archaeal clades (*Methanobrevibacter ruminantium* and relatives and *Methanocorpusculum* and relatives) in equine faeces has previously been reported [22], [54], and these two clades dominated the archaeal microbiome of all horses in the present study (Figure S2). Analysis of the archaeal community of the horses did not show clustering by diet-group and none of the archaeal taxa detected were significantly more or less abundant in either of the treatment groups (Figure S3). These results indicate that the archaeal community structure was not influenced by the change in diet. This finding is aligned with the physiology of the archaeal organisms, which rely on the hydrogen produced by cellulolytic and other bacterial populations reported in the present study and only indirectly on the type of dietary substrate available to the host. However, this does not preclude that by using greater sample size or sequencing depth, impacts on the archaeal community may have been detected.

In the present study, at least 15 different genera of ciliate protozoa were detected in the faeces of pasture-fed horses (Table S2C), of which the genera *Spirodinium* and *Triadinium* were the most abundant. In contrast, previous microscopy studies have reported that the highest percentage composition of ciliates identified in the faeces of horses, were of the genera *Bundleia*, *Blepharocorys*, and *Polymorphella* [55], [56], [57]. The faecal samples in these studies were collected from racehorses that were fed a mixed diet comprised of grain and forages, or from horses with an unknown dietary history; whereas the faecal samples in the present study were from horses fed exclusively on pasture. Faecal microscopic examination was not used in the present study and the ciliate protozoa were amplified from only 26/72 faecal samples. Hence, it was not possible to determine whether there were differences between the ciliate communities of the horses fed different forage-based diets on Day 0 of the study, which may require further investigation in a large number of samples.

The horses used in the present study were of similar age, sex, height, weight and body condition, and were maintained as cohorts in paddocks on the same farm. This is in contrast to most other studies that sampled heterogeneous populations of horses that differed in their feeding and management [13], [15], [22]. The study was not designed to separate the effects of housing from the dietary effects between Groups A and B prior to Day 0, and the change from a loose-box environment to pasture may have influenced the results. However, faecal microbiota are more likely to be influenced by acute changes in diet than more subtle changes in housing. Although the sample size for each group was small (n = 6), it was similar to sample sizes used in previous 454 pyrosequencing-based studies investigating the hindgut microbiome [13], [22].

The faecal samples in the present study were collected carefully from the faecal mass and snap frozen within two minutes of defecation, thereby limiting the possibility of environmental contamination. A number of studies have demonstrated a strong inter-relationship between the microbial communities in the distal region of the hindgut (colon) and faeces [15], [58], [59]. This inter-relationship of microbial communities along the hindgut was reported to be greatest between samples from the right dorsal colon and the rectum/faeces [16]. Therefore, it is reasonable to suggest that the changes observed in the microbial populations of the faecal samples in the present study may be representative of changes occurring in the distal regions of the hindgut. The inter-variation between Groups A and B was higher than intra-variation, as demonstrated by the clustering of horses by treatment group on Day 0 (Figure 4), which allowed the identification of differences between the faecal microbiota of horses fed two different forage-based diets using a sample size of six horses per group.

## Conclusions

The findings of the present study indicate that the faecal bacterial community of yearling horses is highly diverse and the relative abundances of individual taxa change rapidly in response to changes in diet. The faecal microbiota of horses on a conserved forage-grain diet were similar to that of horses fed pasture in terms of species richness and diversity, and the structure of the archaeal communities, but differed significantly in terms of the relative abundances of distinct bacterial families and genera. It is possible that daily changes in pasture composition affect the faecal microbiota of horses and this requires further investigation.

## Supporting Information

Figure S1 **Rarefaction curves for bacterial communities in the faeces of individual horses in Groups A and B at two time-points.** The rarefaction curves show the number of observed species against the depth of sequencing of bacterial communities in the faecal samples from individual horses in Groups A and B on Day 0 (panel A) and Day 4 (panel B). The minimum depth of sequencing per sample for the bacterial group was 1000 sequence reads per sample.
(TIF)

Figure S2 **Relative abundance of archaeal clades in the faecal microbial community of horses.** The chart shows the median relative abundance of archaeal clades in the faeces of horses in Groups A and B, and indicates the dominance of two clades; *Methanocorpusculum* and relatives and *Methanobrevibacter ruminantium* and relatives. The colours in the figure legend show the archaeal clades with median relative abundances >15%.
(TIF)

Figure S3 **Principal coordinate plot for data on the archaeal community structure in the faeces of horses across all sampling days based on Bray-Curtis dissimilarity.** The plot illustrates the similarities in the faecal archaeal communities in the faeces of horses in Groups A and B on six sampling time-points over a period of three weeks. Clustering of horses by diet was not observed on Day 0, and no clustering was seen when horses were fed pasture (Diet P) from Days 4–21, with 84% of the variation explained by PC1.
(TIF)

Figure S4 **3-dimensional plot of bacterial communities in all 72 samples.** The graph contains the data of the bacterial communities in the faeces of horses in Groups A and B at six time-points over the 3-week period. The graph indicates that the horses fed pasture (Diet P, green), from both Groups A (Days 4–21) and B (Days 0–21), clustered separately from the horses fed ensiled conserved forage-grain (Diet F, red) on Day 0.
(TIF)

Table S1 **Dry matter content and nutrient composition of the diets.** A) Nutrient composition of the ensiled lucerne and cracked maize feed (Diet F) that was provided to the horses of Group A prior to Day 0. The nutrient composition is given on a Dry Matter basis, as provided by the manufacturers (Fiber Fresh Feeds Ltd., Reporoa, New Zealand). B) Nutrient composition (Dry Matter basis) of the standard New Zealand rye-clover mixed pasture (Diet P) that was available to Group B horses prior to Day 0 of the study, and Group A and B horses during the study (Days 0–21).
(PDF)

Table S2 **The faecal microbiome of New Zealand Thoroughbred yearling horses fed Diet F (ensiled-forage-grain diet) and Diet P (rye-clover pasture) during the study.** A) The faecal bacterial community, B) The faecal archaeal community, and C) The faecal ciliate protozoal community. The bacterial, archaeal, and ciliate protozoal taxa identified in the faeces of horses in the present study are listed in the table according to the taxonomic ranks assigned using the Greengenes database (version gg_13_5).
(PDF)

Table S3 **Comparison of the relative abundances of bacterial taxa (genus-level) in the faeces of Group A and B horses on Days 0 and 4 of the study.** A) Bacterial genera in the faeces of Group A (fed Diet F) and B horses (fed Diet P) on Day 0 and Day 4 (both groups fed Diet P). B) Bacterial genera in the faeces of Group A horses on Day 0 (fed Diet F) and Day 4 (fed Diet P). C) Bacterial genera in the faeces of Group B horses on Days 0 and 4 (fed Diet P).
(PDF)

Table S4 **Comparison of the relative abundances of archaeal taxa (clade-level) in the faeces of Group A and B horses on Days 0 and 4 of the study.** A) Group A horses fed two different diets in Day 0 (Diet F) and Day 4 (Diet P). B) Group B horses fed pasture (Diet P) on Days 0 and 4. C) Group A and B horses fed pasture (Diet P) on Day 4.
(PDF)
