# Supplementary figures and images for: Faecal Microbiota of Forage-Fed Horses in New Zealand and the Population Dynamics of Microbial Communities following Dietary Change
Source: PLoS One. 2014 Nov 10;9(11):e112846. doi: 10.1371/journal.pone.0112846 (PMC4226576; doi:10.1371/journal.pone.0112846)

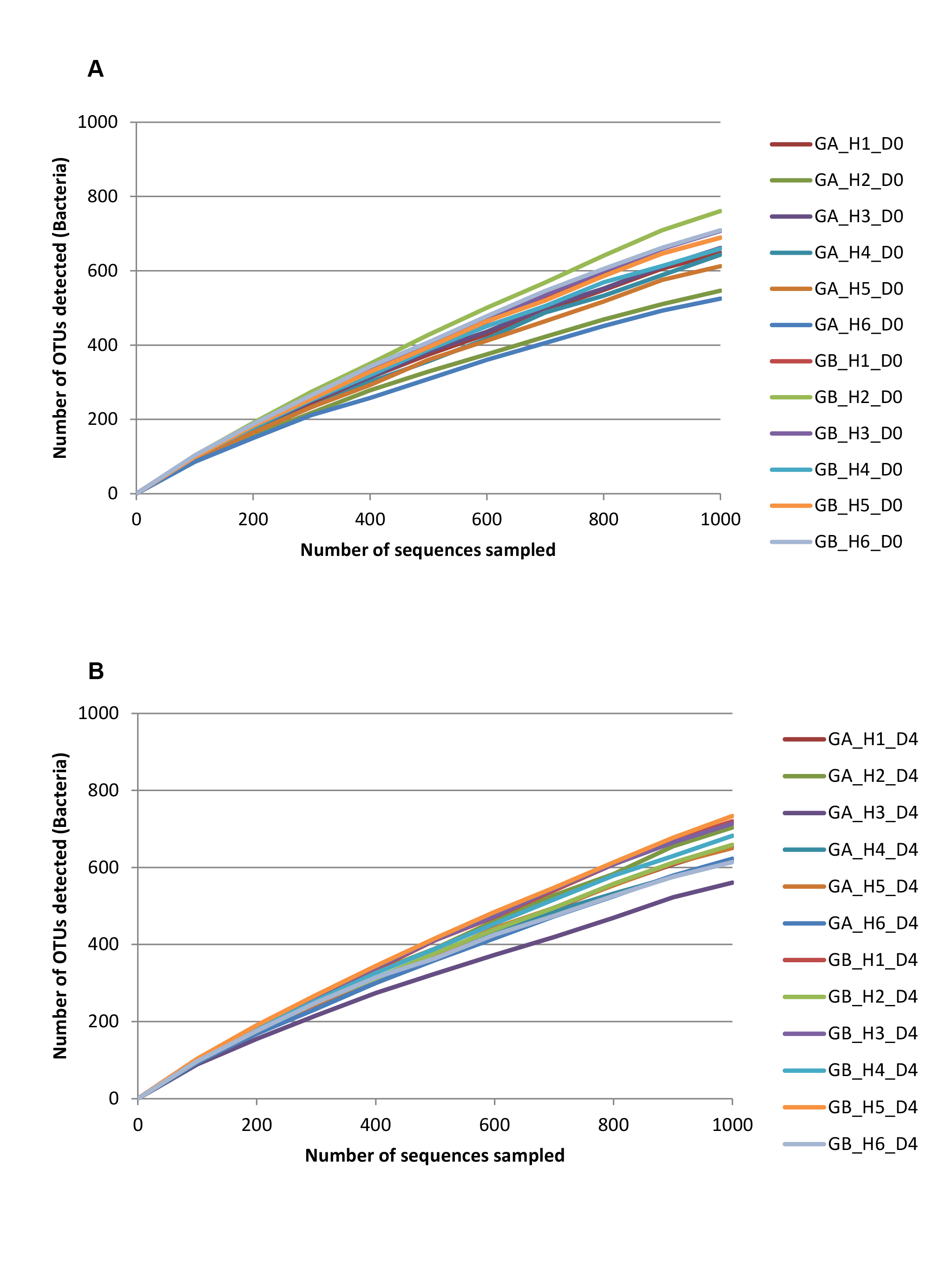

Supplement: Figure S1 — Rarefaction curves for bacterial communities in the faeces of individual horses in Groups A and B at two time-points. The rarefaction curves show the number of observed species against the depth of sequencing of bacterial communities in the faecal samples from individual horses in Groups A and B on Day 0 (panel A) and Day 4 (panel B). The minimum depth of sequencing per sample for the bacterial group was 1000 sequence reads per sample. (TIF) [file pone.0112846.s001.tif]

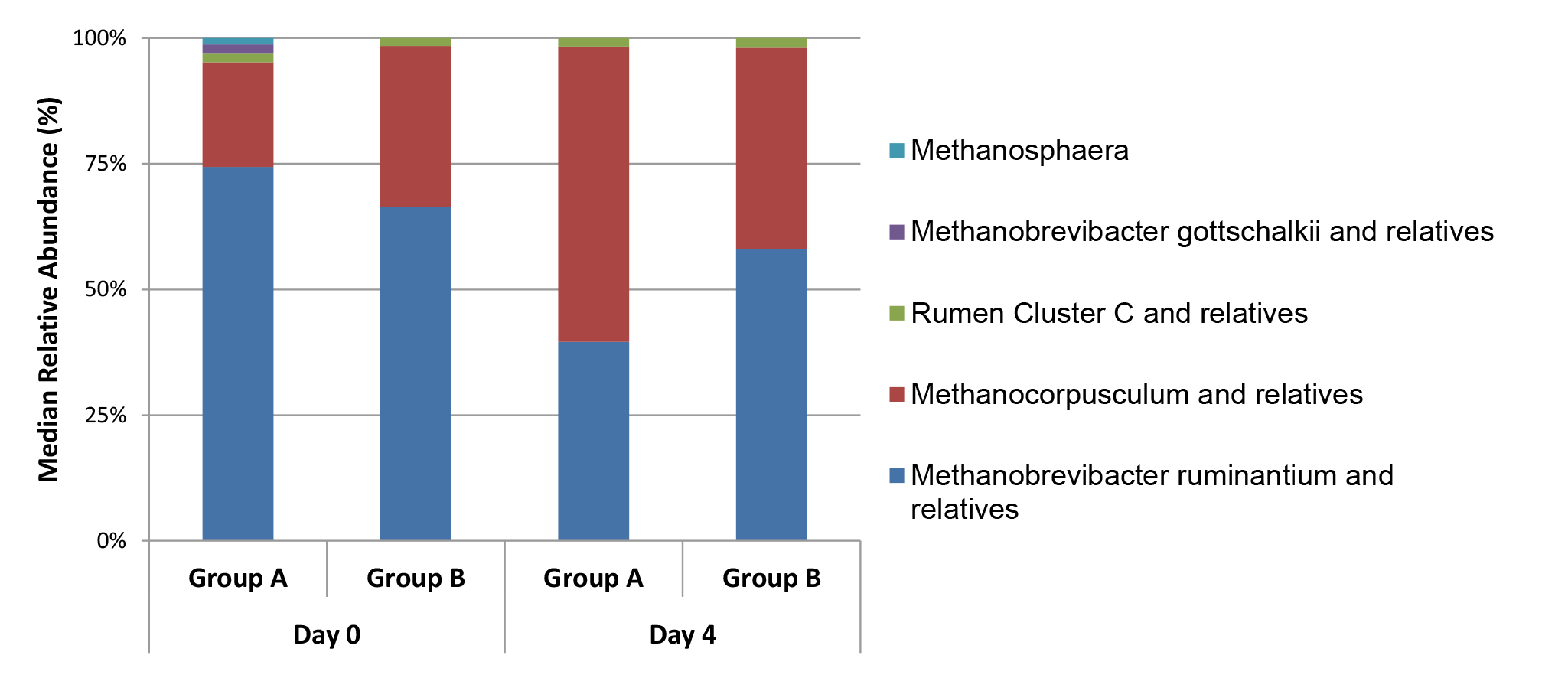

Supplement: Figure S2 — Relative abundance of archaeal clades in the faecal microbial community of horses. The chart shows the median relative abundance of archaeal clades in the faeces of horses in Groups A and B, and indicates the dominance of two clades; Methanocorpusculum and relatives and Methanobrevibacter ruminantium and relatives. The colours in the figure legend show the archaeal clades with median relative abundances >15%. (TIF) [file pone.0112846.s002.tif]

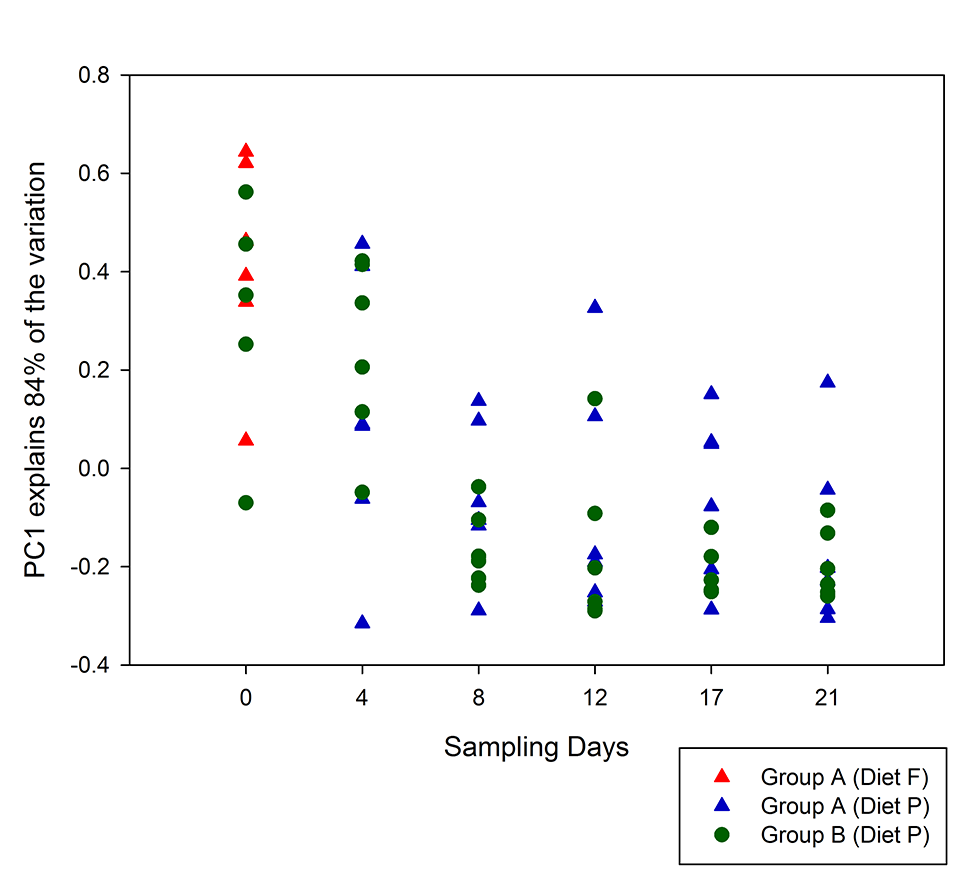

Supplement: Figure S3 — Principal coordinate plot for data on the archaeal community structure in the faeces of horses across all sampling days based on Bray-Curtis dissimilarity. The plot illustrates the similarities in the faecal archaeal communities in the faeces of horses in Groups A and B on six sampling time-points over a period of three weeks. Clustering of horses by diet was not observed on Day 0, and no clustering was seen when horses were fed pasture (Diet P) from Days 4–21, with 84% of the variation explained by PC1. (TIF) [file pone.0112846.s003.tif]

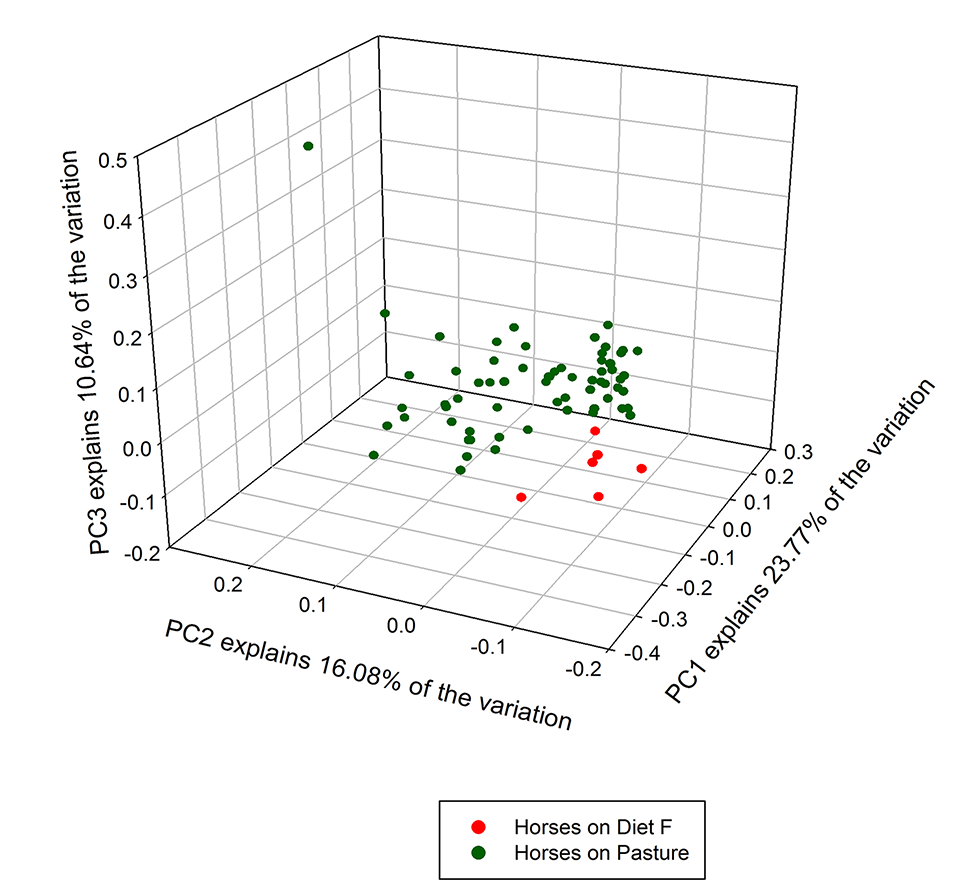

Supplement: Figure S4 — 3-dimensional plot of bacterial communities in all 72 samples. The graph contains the data of the bacterial communities in the faeces of horses in Groups A and B at six time-points over the 3-week period. The graph indicates that the horses fed pasture (Diet P, green), from both Groups A (Days 4–21) and B (Days 0–21), clustered separately from the horses fed ensiled conserved forage-grain (Diet F, red) on Day 0. (TIF) [file pone.0112846.s004.tif]
