# Supplementary material for: Faecal Microbiota of Forage-Fed Horses in New Zealand and the Population Dynamics of Microbial Communities following Dietary Change
Source: PLoS One. 2014 Nov 10;9(11):e112846. doi: 10.1371/journal.pone.0112846 (PMC4226576; doi:10.1371/journal.pone.0112846)
Supplement: Table S1 — Dry matter content and nutrient composition of the diets. A) Nutrient composition of the ensiled lucerne and cracked maize feed (Diet F) that was provided to the horses of Group A prior to Day 0. The nutrient composition is given on a Dry Matter basis, as provided by the manufacturers (Fiber Fresh Feeds Ltd., Reporoa, New Zealand). B) Nutrient composition (Dry Matter basis) of the standard New Zealand rye-clover mixed pasture (Diet P) that was available to Group B horses prior to Day 0 of the study, and Group A and B horses during the study (Days 0–21). (PDF) [file pone.0112846.s005.pdf]

Table S1. Dry matter content and nutrient composition of the diets.

A) Nutrient composition of the ensiled lucerne and cracked maize feed (Diet F) that was provided to the horses of Group A prior to Day 0. The nutrient composition is given on a Dry Matter basis, as provided by the manufacturers (Fiber Fresh Feeds Ltd., Reporoa, New Zealand).

	Diet F
	Group A horses
Dry matter, g/100g	59.30
Crude protein, g/100g	11.70
Crude fibre, g/100g	14.80
Crude fat, g/100g	3.00
Calcium, g/100g	1.00
Phosphorus, g/100g	0.52
Selenium, mg/100g	0.03
Digestible energy (MJ/kg)	11.98

B) Nutrient composition (Dry Matter basis) of the standard New Zealand rye-clover mixed pasture (Diet P) that was available to Group B horses prior to Day 0 of the study, and Group A and B horses during the study (Days 0-21).

	Diet P	
	Group A Horses	Group B Horses
Dry Matter %	29.5	28.5
Ash %	7.9	7.2
Fat %	2.2	2.3
Crude Protein %	13.2	11.1
Neutral Detergent Fibre (NDF) %	52.8	52.3
Acid Detergent Fibre (ADF) %	25.9	27.0
Lignin %	3.1	3.2
Starch %	1.1	0.8
Gross Energy kJ/g	18.0	18.0
