# Supplementary material for: Faecal Microbiota of Forage-Fed Horses in New Zealand and the Population Dynamics of Microbial Communities following Dietary Change
Source: PLoS One. 2014 Nov 10;9(11):e112846. doi: 10.1371/journal.pone.0112846 (PMC4226576; doi:10.1371/journal.pone.0112846)
Supplement: Table S2 — The faecal microbiome of New Zealand Thoroughbred yearling horses fed Diet F (ensiled-forage-grain diet) and Diet P (rye-clover pasture) during the study. A) The faecal bacterial community, B) The faecal archaeal community, and C) The faecal ciliate protozoal community. The bacterial, archaeal, and ciliate protozoal taxa identified in the faeces of horses in the present study are listed in the table according to the taxonomic ranks assigned using the Greengenes database (version gg_13_5). (PDF) [file pone.0112846.s006.pdf]

Table S2. The faecal microbiome of New Zealand Thoroughbred yearling horses fed Diet F (ensiled-forage-grain diet) and Diet P (rye-clover pasture) during the study.

The bacterial, archaeal, and ciliate protozoal taxa identified in the faeces of horses in the present study are listed in the table according to the taxonomic ranks assigned using the Greengenes database (version gg_13_5).

A. The faecal bacterial community

Domain	Phylum	Class	Order	Family	Genus	Species
Bacteria	Actinobacteria	Acidimicrobia	Acidimicrobiales	unclassified	unclassified	unclassified
		Actinobacteria	Actinomycetales	Microbacteriaceae	unclassified	unclassified
					Agromyces	unclassified
					Curtobacterium	unclassified
					Microbacterium	unclassified
					Mycetocola	unclassified
					Salinibacterium	unclassified
				Micrococcaceae	unclassified	unclassified
					Arthrobacter	unclassified
				Mycobacteriaceae	*Mycobacterium*	unclassified
				Nocardiaceae	*Rhodococcus*	unclassified
					Rhodococcus	*R. fascians*
				Nocardioidaceae	unclassified	unclassified
				Streptomycetaceae	unclassified	unclassified
					Streptomyces	unclassified
				Williamsiaceae	*Williamsia*	unclassified
		Coriobacteriia	Coriobacteriales	Coriobacteriaceae	unclassified	unclassified
					Adlercreutzia	unclassified
					Eggerthella	unclassified
		OPB41	unclassified	unclassified	unclassified	unclassified
	Armatimonadetes	SJA-176	RB046	unclassified	unclassified	unclassified
	Bacteroidetes	Bacteroidia	Bacteroidales	unclassified	unclassified	unclassified
				BS11	unclassified	unclassified
				Bacteroidaceae	unclassified	unclassified
					5-7N15	unclassified
					BF311	unclassified
					Bacteroides	unclassified
						B. coprophilus
						B. coprosuis
						B. plebeius
						B. uniformis

Domain	Phylum	Class	Order	Family	Genus	Species
				Marinilabiaceae	unclassified	unclassified
				Porphyromonadaceae	*Paludibacter*	unclassified
					Parabacteroides	unclassified
					Parabacteroides	*P. distasonis*
				Prevotellaceae	unclassified	unclassified
					Prevotella	unclassified
						P. copri
						P. melaninogenica
						P. stercorea
				RF16	unclassified	unclassified
				Rikenellaceae	unclassified	unclassified
					PW3	unclassified
				S24-7	unclassified	unclassified
				[Barnesiellaceae]	unclassified	unclassified
				[Odoribacteraceae]	*Butyricimonas*	unclassified
				[Paraprevotellaceae]	unclassified	unclassified
					CF231	unclassified
					Paraprevotella	unclassified
					YRC22	unclassified
					[Prevotella]	unclassified
				p-2534-18B5	unclassified	unclassified
		[Saprospirae]	[Saprospirales]	Chitinophagaceae	unclassified	unclassified
	Chloroflexi	Anaerolineae	Anaerolineales	Anaerolinaceae	SHD-231	unclassified
			GCA004	unclassified	unclassified	unclassified
		Dehalococcoidetes	Dehalococcoidales	Dehalococcoidaceae	unclassified	unclassified
		Thermomicrobia	JG30-KF-CM45	unclassified	unclassified	unclassified
	Cyanobacteria	4C0d-2	YS2	unclassified	unclassified	unclassified
		Chloroplast	Streptophyta	unclassified	unclassified	unclassified
	Elusimicrobia	Elusimicrobia	Elusimicrobiales	Elusimicrobiaceae	unclassified	unclassified
			unclassified	unclassified	*Elusimicrobium*	unclassified
		Endomicrobia	unclassified	unclassified	unclassified	unclassified
	Fibrobacteres	Fibrobacteria	Fibrobacterales	Fibrobacteraceae	*Fibrobacter*	*F. succinogenes*
	Firmicutes	Bacilli	Bacillales	Bacillaceae	*Bacillus*	*B. longiquaesitum*
				Paenibacillaceae	*Paenibacillus*	unclassified
				Planococcaceae	*Sporosarcina*	unclassified
			Lactobacillales	Aerococcaceae	unclassified	unclassified
				Carnobacteriaceae	*Trichococcus*	unclassified
				Lactobacillaceae	*Lactobacillus*	unclassified
						L. salivarius
		Clostridia	unclassified	unclassified	unclassified	unclassified
			Clostridiales	unclassified	unclassified	unclassified

Domain	Phylum	Class	Order	Family	Genus	Species
				Caldicoprobacteraceae	*Caldicoprobacter*	unclassified
				Christensenellaceae	unclassified	unclassified
				Clostridiaceae	unclassified	unclassified
					02d06	unclassified
					Clostridium	unclassified
						C. butyricum
					Geosporobacter Thermotalea	unclassified
					Sarcina	unclassified
				Dehalobacteriaceae	unclassified	unclassified
				EtOH8	unclassified	unclassified
				Eubacteriaceae	*Anaerofustis*	unclassified
					Pseudoramibacter Eubacterium	unclassified
				Lachnospiraceae	unclassified	unclassified
					Anaerostipes	unclassified
					Blautia	unclassified
					Blautia	*B. producta*
					Butyrivibrio	unclassified
					Coprococcus	unclassified
						C. catus
						C. eutactus
					Dorea	unclassified
						D. formicigenerans
					Epulopiscium	unclassified
					Lachnobacterium	unclassified
					Lachnospira	unclassified
					Moryella	unclassified
					Pseudobutyrivibrio	unclassified
					Roseburia	unclassified
						R. faecis
					[Ruminococcus]	unclassified
						R. gnavus
						R. torques
				Peptococcaceae	unclassified	unclassified
					Desulfotomaculum	unclassified
					rc4-4	unclassified
				Peptostreptococcaceae	unclassified	unclassified
				Ruminococcaceae	unclassified	unclassified
					Ethanoligenens	unclassified
					Faecalibacterium	unclassified
						F. prausnitzii
					Oscillospira	unclassified

Domain	Phylum	Class	Order	Family	Genus	Species
					Ruminococcus	unclassified
						R. bromii
						R. flavefaciens
				Syntrophomonadaceae	*Syntrophomonas*	unclassified
				Veillonellaceae	unclassified	unclassified
					Anaerovibrio	unclassified
					Phascolarctobacterium	unclassified
					Selenomonas	unclassified
					Succiniclasticum	unclassified
				[Acidaminobacteraceae]	WH1-8	unclassified
				[Mogibacteriaceae]	unclassified	unclassified
					Anaerovorax	unclassified
					Mogibacterium	unclassified
		Erysipelotrichi	Erysipelotrichales	Erysipelotrichaceae	unclassified	unclassified
					Bulleidia	unclassified
						p-1630-c5
					Coprobacillus	unclassified
					L7A E11	unclassified
					RFN20	unclassified
					Sharpea	unclassified
					[Eubacterium]	unclassified
						E. biforme
						E. cylindroides
					cc 115	unclassified
					p-75-a5	unclassified
	Fusobacteria	Fusobacteriia	Fusobacteriales	Fusobacteriaceae	Fusobacterium	unclassified
	LD1	unclassified	unclassified	unclassified	unclassified	unclassified
	Lentisphaerae	[Lentisphaeria]	Victivallales	Victivallaceae	unclassified	unclassified
			Z20	R4-45B	unclassified	unclassified
	Planctomycetes	Planctomycetia	Pirellulales	Pirellulaceae	unclassified	unclassified
	Proteobacteria	Alphaproteobacteria		unclassified	unclassified	unclassified
			RF32	unclassified	unclassified	unclassified
			Rhizobiales	Rhizobiaceae	*Agrobacterium*	unclassified
			Rickettsiales	unclassified	unclassified	unclassified
		Betaproteobacteria	Burkholderiales	Alcaligenaceae	*Sutterella*	unclassified
				Oxalobacteraceae	*Cupriavidus*	unclassified
					Oxalobacter	unclassified
						O. formigenes
			Neisseriales	Neisseriaceae	*Neisseria*	unclassified
			Tremblayales	unclassified	unclassified	unclassified
		Deltaproteobacteria	Desulfovibrionales	Desulfovibrionaceae	unclassified	unclassified

Domain	Phylum	Class	Order	Family	Genus	Species
					Desulfovibrio	unclassified
			GMD14H09	unclassified	unclassified	unclassified
		Epsilonproteobacteria	Campylobacterales	Campylobacteraceae	*Campylobacter*	unclassified
		Gammaproteobacteria	Aeromonadales	Succinivibrionaceae	unclassified	unclassified
					Succinivibrio	unclassified
			Pasteurellales	Pasteurellaceae	*Aggregatibacter*	unclassified
	Spirochaetes	MVP-15	PL-11B10	unclassified	unclassified	unclassified
		Spirochaetes	Sphaerochaetales	Sphaerochaetaceae	unclassified	unclassified
					Sphaerochaeta	unclassified
			Spirochaetales	Spirochaetaceae	*Treponema*	unclassified
	Synergistetes	Synergistia	Synergistales	unclassified	unclassified	unclassified
				Dethiosulfovibrionaceae	unclassified	unclassified
					TG5	unclassified
				Synergistaceae	unclassified	unclassified
					Candidatus Tammella	unclassified
					vadinCA02	unclassified
	TM7	TM7-3	CW040	unclassified	unclassified	unclassified
			EW055	unclassified	unclassified	unclassified
	Tenericutes	Mollicutes	Acholeplasmatales	unclassified	unclassified	unclassified
				Anaeroplasmataceae	unclassified	unclassified
					Anaeroplasma	unclassified
			Mycoplasmatales	Mycoplasmataceae	unclassified	unclassified
			RF39	unclassified	unclassified	unclassified
		RF3	ML615J-28	unclassified	unclassified	unclassified
	unclassified	unclassified	unclassified	unclassified	unclassified	unclassified
	Verrucomicrobia	unclassified	unclassified	unclassified	unclassified	unclassified
		Opitutae	HA64	unclassified	unclassified	unclassified
		Verruco-5	WCHB1-41	RFP12	unclassified	unclassified
		Verrucomicrobiae	Verrucomicrobiales	Verrucomicrobiaceae	*Akkermansia*	unclassified

B. The faecal archaeal community

Domain	Phylum	Class	Order	Family	Genus	Clade
Archaea	Crenarchaeota	Thermoprotei			*Sulfolobus* and relatives	unclassified
	Euryarchaeota	Methanobacteria	Methanobacteriales	Methanobacteriaceae	*Methanobacterium*	unclassified
					Methanobrevibacter	*Methanobrevibacter arboriphilus* and relatives
						Methanobrevibacter gottschalkii and relatives
						Methanobrevibacter ruminantium and relatives
					Methanosphaera	unclassified
		Methanomicrobia	Methanomicrobiales		*Methanocorpusculum* and relatives	unclassified
			Methanosarcinales	Methanosarcinaceae	*Methanimicrococcus*	unclassified
					Methanosarcina and relatives	unclassified
		Thermoplasmata		Thermoplasmatales	Rumen Cluster C and relatives	unclassified

C. The faecal ciliate protozoal community

Domain	Kingdom	Super-phylum	Phylum	Class	Sub-class	Genus
Eukarya	Chromalveolata	Alveolata	Ciliophora	Litostomatea	Haptoria	*Didinium*
						Epiphyllum
						Pelagodileptus
					Trichostomatia	*Blepharocorys*
						Bundleia
						Cochliatoxum
						Cycloposthium
						Diplodinium
						Entodinium
						Eremoplastron
						Isotricha
						Polydiniella
						Spirodinium
						Triadinium
			unclassified		unclassified	unclassified
