# Supplementary material for: Faecal Microbiota of Forage-Fed Horses in New Zealand and the Population Dynamics of Microbial Communities following Dietary Change
Source: PLoS One. 2014 Nov 10;9(11):e112846. doi: 10.1371/journal.pone.0112846 (PMC4226576; doi:10.1371/journal.pone.0112846)
Supplement: Table S3 — Comparison of the relative abundances of bacterial taxa (genus-level) in the faeces of Group A and B horses on Days 0 and 4 of the study. A) Bacterial genera in the faeces of Group A (fed Diet F) and B horses (fed Diet P) on Day 0 and Day 4 (both groups fed Diet P). B) Bacterial genera in the faeces of Group A horses on Day 0 (fed Diet F) and Day 4 (fed Diet P). C) Bacterial genera in the faeces of Group B horses on Days 0 and 4 (fed Diet P). (PDF) [file pone.0112846.s007.pdf]

Table S3. Comparison of the relative abundances of bacterial taxa (genus-level) in the faeces of Group A and B horses on Days 0 and 4 of the study.

A) Bacterial genera in the faeces of Group A (fed Diet F) and B horses (fed Diet P) on Day 0 and Day 4 (both groups fed Diet P).

Taxonomic rank within the domain Bacteria Phylum > Class > Order > Family > Genus	Relative abundances on Day 0					Relative abundances on Day 4				
	Group A		Group B			Group A		Group B		
	Median	IQR[a]	Median	IQR[a]	*P* -Value[b]	Median	IQR[a]	Median	IQR[a]	*P* –Value[b]
Actinobacteria > Coriobacteria > Coriobacteriales > Coriobacteriaceae > unclassified	0.001	0.001 - 0.002	0.005	0.002 - 0.015	0.034	0.005	0.004 - 0.007	0.006	0.004 - 0.010	0.623
Armatimonadetes > SJA-176 > RB046 > unclassified > unclassified	0.001	0.000 - 0.001	0.002	0.001 - 0.003	0.134	0.009	0.006 - 0.017	0.002	0.001 - 0.002	0.011
Bacteroidetes > Bacteroidia > Bacteroidales >unclassified > unclassified	0.079	0.053 - 0.100	0.091	0.080 - 0.132	0.688	0.158	0.132 - 0.177	0.134	0.117 - 0.181	0.574
Bacteroidetes > Bacteroidia > Bacteroidales > BS11 > unclassified	0.006	0.001 - 0.012	0.004	0.002 - 0.007	0.871	0.003	0.001 - 0.006	0.007	0.004 - 0.012	0.260
Bacteroidetes > Bacteroidia > Bacteroidales > Bacteroidaceae > BF311	0.001	0.000 - 0.001	0.010	0.009 - 0.014	0.003*	0.007	0.001 - 0.021	0.011	0.008 - 0.011	0.935
Bacteroidetes > Bacteroidia > Bacteroidales > Bacteroidaceae > *Bacteroides*	0.001	0.000 - 0.005	0.001	0.000 - 0.002	1.000	0.002	0.001 - 0.007	0.003	0.002 - 0.003	0.869
Bacteroidetes > Bacteroidia > Bacteroidales > Porphyromonadaceae > *Paludibacter*	0.000	0.000 - 0.001	0.001	0.000 - 0.001	0.476	0.000	0.000 - 0.000	0.001	0.000 - 0.001	0.390
Bacteroidetes > Bacteroidia > Bacteroidales > Porphyromonadaceae > *Parabacteroides*	0.000	0.000 - 0.000	0.000	0.000 - 0.000	0.317	0.004	0.000 - 0.024	0.000	0.000 - 0.000	0.058
Bacteroidetes > Bacteroidia > Bacteroidales > Prevotellaceae > unclassified	0.000	0.000 - 0.003	0.001	0.001 - 0.002	0.402	0.001	0.001 - 0.002	0.000	0.000 - 0.001	0.014
Bacteroidetes > Bacteroidia > Bacteroidales > Prevotellaceae > *Prevotella*	0.010	0.003 - 0.024	0.027	0.020 - 0.039	0.172	0.028	0.020 - 0.065	0.035	0.026 - 0.048	1.000
Bacteroidetes > Bacteroidia > Bacteroidales > RF16 > unclassified	0.002	0.000 - 0.003	0.002	0.000 - 0.002	0.741	0.002	0.001 - 0.004	0.001	0.000 - 0.002	0.138
Bacteroidetes > Bacteroidia > Bacteroidales > S24-7 > unclassified	0.007	0.004 - 0.011	0.003	0.002 - 0.003	0.050	0.002	0.001 - 0.005	0.003	0.003 - 0.006	0.410
Bacteroidetes > Bacteroidia > Bacteroidales > [Paraprevotellaceae] > unclassified	0.006	0.002 - 0.012	0.010	0.007 - 0.015	0.261	0.007	0.005 - 0.015	0.012	0.011 - 0.016	0.148
Bacteroidetes > Bacteroidia > Bacteroidales > [Paraprevotellaceae] > CF231	0.002	0.000 - 0.003	0.012	0.008 - 0.022	0.004*	0.004	0.002 - 0.011	0.011	0.006 - 0.014	0.091
Bacteroidetes > Bacteroidia > Bacteroidales > [Paraprevotellaceae] > YRC22	0.040	0.006 - 0.063	0.043	0.037 - 0.067	0.470	0.023	0.017 - 0.025	0.031	0.022 - 0.050	0.336
Bacteroidetes > Bacteroidia > Bacteroidales > [Paraprevotellaceae] > [Prevotella]	0.005	0.002 - 0.010	0.006	0.001 - 0.010	0.872	0.003	0.001 - 0.005	0.003	0.001 - 0.008	0.806
Cyanobacteria > 4C0d-2 > YS2 > unclassified > unclassified	0.004	0.001 - 0.004	0.001	0.000 - 0.002	0.121	0.002	0.000 - 0.002	0.002	0.001 - 0.007	0.404
Fibrobacteres > Fibrobacteria > Fibrobacterales > Fibrobacteraceae > *Fibrobacter*	0.003	0.002 - 0.005	0.005	0.003 - 0.009	0.197	0.008	0.004 - 0.010	0.010	0.008 - 0.017	0.199
Firmicutes > Clostridia > unclassified > unclassified > unclassified	0.001	0.000 - 0.003	0.000	0.000 - 0.001	0.281	0.002	0.000 - 0.003	0.002	0.001 - 0.002	0.867
Firmicutes > Clostridia > Clostridiales > unclassified > unclassified	0.112	0.103 - 0.131	0.163	0.149 - 0.181	0.003*	0.151	0.130 - 0.167	0.146	0.131 - 0.164	0.872
Firmicutes > Clostridia > Clostridiales > Christensenellaceae > unclassified	0.007	0.004 - 0.010	0.005	0.004 - 0.006	0.462	0.004	0.002 - 0.007	0.006	0.003 - 0.007	0.685
Firmicutes > Clostridia > Clostridiales > Clostridiaceae > unclassified	0.006	0.003 - 0.017	0.006	0.004 - 0.008	0.872	0.005	0.004 - 0.005	0.006	0.003 - 0.012	0.808
Firmicutes > Clostridia > Clostridiales > Clostridiaceae > *Clostridium*	0.010	0.007 - 0.015	0.010	0.006 - 0.014	0.747	0.006	0.005 - 0.008	0.013	0.007 - 0.015	0.335
Firmicutes > Clostridia > Clostridiales > Eubacteriaceae > *Pseudoramibacter_Eubacterium*	0.001	0.000 - 0.002	0.002	0.000 - 0.004	0.359	0.006	0.004 - 0.007	0.003	0.002 - 0.005	0.166
Firmicutes > Clostridia > Clostridiales > Lachnospiraceae > unclassified	0.098	0.067 - 0.139	0.188	0.157 - 0.238	0.006*	0.173	0.151 - 0.195	0.179	0.167 - 0.208	0.521
Firmicutes > Clostridia > Clostridiales > Lachnospiraceae > *Blautia*	0.006	0.003 - 0.008	0.012	0.007 - 0.015	0.172	0.004	0.002 - 0.009	0.007	0.005 - 0.009	0.374
Firmicutes > Clostridia > Clostridiales > Lachnospiraceae > *Coprococcus*	0.005	0.005 - 0.007	0.011	0.008 - 0.011	0.062	0.017	0.011 - 0.021	0.011	0.008 - 0.012	0.062
Firmicutes > Clostridia > Clostridiales > Ruminococcaceae > unclassified	0.395	0.387 - 0.413	0.281	0.227 - 0.293	0.016	0.205	0.171 - 0.220	0.226	0.158 - 0.290	0.748
Firmicutes > Clostridia > Clostridiales > Ruminococcaceae > *Oscillospira*	0.015	0.013 - 0.019	0.008	0.006 - 0.008	0.044	0.009	0.003 - 0.010	0.006	0.004 - 0.011	0.936
Firmicutes > Clostridia > Clostridiales > Ruminococcaceae > *Ruminococcus*	0.045	0.028 - 0.058	0.019	0.015 - 0.030	0.054	0.025	0.018 - 0.032	0.024	0.017 - 0.037	1.000
Firmicutes > Clostridia > Clostridiales > [Mogibacteriaceae] > unclassified	0.009	0.008 - 0.011	0.011	0.007 - 0.012	0.872	0.017	0.012 - 0.033	0.015	0.012 - 0.016	0.331
Firmicutes > Clostridia > Clostridiales > [Mogibacteriaceae] > *Mogibacterium*	0.006	0.001 - 0.013	0.005	0.004 - 0.005	0.416	0.006	0.005 - 0.008	0.005	0.002 - 0.009	0.420
Firmicutes > Erysipelotrichi > Erysipelotrichales > Erysipelotrichaceae > RFN20	0.008	0.003 - 0.013	0.003	0.003 - 0.008	0.135	0.010	0.008 - 0.015	0.002	0.002 - 0.004	0.004*
Firmicutes > Erysipelotrichi > Erysipelotrichales > Erysipelotrichaceae > p-75-a5	0.004	0.000 - 0.006	0.002	0.001 - 0.002	0.329	0.004	0.002 - 0.008	0.001	0.000 - 0.002	0.059
Planctomycetes > Planctomycetia > Pirellulales > Pirellulaceae > unclassified	0.001	0.000 - 0.003	0.000	0.000 - 0.000	0.181	0.001	0.000 - 0.002	0.001	0.000 - 0.001	0.445
Proteobacteria > Alphaproteobacteria > RF32 > unclassified > unclassified	0.000	0.000 - 0.001	0.000	0.000 - 0.001	1.000	0.001	0.000 - 0.001	0.000	0.000 - 0.001	0.575
Proteobacteria > Betaproteobacteria > Tremblayales > unclassified > unclassified	0.000	0.000 - 0.000	0.000	0.000 - 0.001	0.138	0.000	0.000 - 0.000	0.001	0.000 - 0.002	0.209
Proteobacteria > Gammaproteobacteria > Aeromonadales > Succinivibrionaceae > unclassified	0.000	0.000 - 0.002	0.000	0.000 - 0.000	0.139	0.000	0.000 - 0.000	0.000	0.000 - 0.000	n/p
Spirochaetes > Spirochaetes > Spirochaetales > Spirochaetaceae > *Treponema*	0.005	0.004 - 0.006	0.002	0.002 - 0.004	0.101	0.002	0.001 - 0.005	0.005	0.003 - 0.007	0.078
Synergistetes > Synergistia > Synergistales > Synergistaceae > unclassified	0.000	0.000 - 0.000	0.000	0.000 - 0.000	0.317	0.000	0.000 - 0.000	0.000	0.000 - 0.000	0.902
Other Genera <1%	0.028	0.022 - 0.029	0.033	0.029 - 0.036	0.077	0.030	0.029 - 0.032	0.032	0.026 - 0.038	0.518

[a] IQR – Interquartile range

[b] Level of statistical significance after Bonferroni adjustment for multiple comparisons *P* = 0.001

*Differences between Groups A and B

n/p – no *P*-value

B) Bacterial genera in the faeces of Group A horses on Day 0 (fed Diet F) and Day 4 (fed Diet P).

Taxonomic rank within the domain Bacteria<br>Phylum > Class > Order > Family > Genus	Relative abundances of Group A horses<br>Day 0		Day 4		
	**Median**	**IQR[a]**	**Median**	**IQR[a]**	***P* -Value[b]**
Actinobacteria > Coriobacteria > Coriobacteriales > Coriobacteriaceae > unclassified	0.001	0.001 - 0.002	0.005	0.004 - 0.007	0.050
Armatimonadetes > SJA-176 > RB046 > unclassified > unclassified	0.001	0.000 - 0.001	0.009	0.006 - 0.017	0.005*
Bacteroidetes > Bacteroidia > Bacteroidales >unclassified > unclassified	0.079	0.053 - 0.100	0.158	0.132 - 0.177	0.109
Bacteroidetes > Bacteroidia > Bacteroidales > BS11 > unclassified	0.006	0.001 - 0.012	0.003	0.001 - 0.006	0.809
Bacteroidetes > Bacteroidia > Bacteroidales > Bacteroidaceae > BF311	0.001	0.000 - 0.001	0.007	0.001 - 0.021	0.085
Bacteroidetes > Bacteroidia > Bacteroidales > Bacteroidaceae > *Bacteroides*	0.001	0.000 - 0.005	0.002	0.001 - 0.007	0.413
Bacteroidetes > Bacteroidia > Bacteroidales > Porphyromonadaceae > *Paludibacter*	0.000	0.000 - 0.001	0.000	0.000 - 0.000	0.598
Bacteroidetes > Bacteroidia > Bacteroidales > Porphyromonadaceae > *Parabacteroides*	0.000	0.000 - 0.000	0.004	0.000 - 0.024	0.153
Bacteroidetes > Bacteroidia > Bacteroidales > Prevotellaceae > unclassified	0.000	0.000 - 0.003	0.001	0.001 - 0.002	0.212
Bacteroidetes > Bacteroidia > Bacteroidales > Prevotellaceae > *Prevotella*	0.010	0.003 - 0.024	0.028	0.020 - 0.065	0.078
Bacteroidetes > Bacteroidia > Bacteroidales > RF16 > unclassified	0.002	0.000 - 0.003	0.002	0.001 - 0.004	0.370
Bacteroidetes > Bacteroidia > Bacteroidales > S24-7 > unclassified	0.007	0.004 - 0.011	0.002	0.001 - 0.005	0.106
Bacteroidetes > Bacteroidia > Bacteroidales > [Paraprevotellaceae] > unclassified	0.006	0.002 - 0.012	0.007	0.005 - 0.015	0.519
Bacteroidetes > Bacteroidia > Bacteroidales > [Paraprevotellaceae] > CF231	0.002	0.000 - 0.003	0.004	0.002 - 0.011	0.169
Bacteroidetes > Bacteroidia > Bacteroidales > [Paraprevotellaceae] > YRC22	0.040	0.006 - 0.063	0.023	0.017 - 0.025	0.749
Bacteroidetes > Bacteroidia > Bacteroidales > [Paraprevotellaceae] > [Prevotella]	0.005	0.002 - 0.010	0.003	0.001 - 0.005	0.332
Cyanobacteria > 4C0d-2 > YS2 > unclassified > unclassified	0.004	0.001 - 0.004	0.002	0.000 - 0.002	0.166
Fibrobacteres > Fibrobacteria > Fibrobacterales > Fibrobacteraceae > *Fibrobacter*	0.003	0.002 - 0.005	0.008	0.004 - 0.010	0.076
Firmicutes > Clostridia > unclassified > unclassified > unclassified	0.001	0.000 - 0.003	0.002	0.000 - 0.003	0.613
Firmicutes > Clostridia > Clostridiales > unclassified > unclassified	0.112	0.103 - 0.131	0.151	0.130 - 0.167	0.078
Firmicutes > Clostridia > Clostridiales > Christensenellaceae > unclassified	0.007	0.004 - 0.010	0.004	0.002 - 0.007	0.318
Firmicutes > Clostridia > Clostridiales > Clostridiaceae > unclassified	0.006	0.003 - 0.017	0.005	0.004 - 0.005	0.571
Firmicutes > Clostridia > Clostridiales > Clostridiaceae > *Clostridium*	0.010	0.007 - 0.015	0.006	0.005 - 0.008	0.092
Firmicutes > Clostridia > Clostridiales > Eubacteriaceae > *Pseudoramibacter_Eubacterium*	0.001	0.000 - 0.002	0.006	0.004 - 0.007	0.004*
Firmicutes > Clostridia > Clostridiales > Lachnospiraceae > unclassified	0.098	0.067 - 0.139	0.173	0.151 - 0.195	0.025
Firmicutes > Clostridia > Clostridiales > Lachnospiraceae > *Blautia*	0.006	0.003 - 0.008	0.004	0.002 - 0.009	0.630
Firmicutes > Clostridia > Clostridiales > Lachnospiraceae > *Coprococcus*	0.005	0.005 - 0.007	0.017	0.011 - 0.021	0.024
Firmicutes > Clostridia > Clostridiales > Ruminococcaceae > unclassified	0.395	0.387 - 0.413	0.205	0.171 - 0.220	0.004*
Firmicutes > Clostridia > Clostridiales > Ruminococcaceae > *Oscillospira*	0.015	0.013 - 0.019	0.009	0.003 - 0.010	0.025
Firmicutes > Clostridia > Clostridiales > Ruminococcaceae > *Ruminococcus*	0.045	0.028 - 0.058	0.025	0.018 - 0.032	0.146
Firmicutes > Clostridia > Clostridiales > [Mogibacteriaceae] > unclassified	0.009	0.008 - 0.011	0.017	0.012 - 0.033	0.024
Firmicutes > Clostridia > Clostridiales > [Mogibacteriaceae] > *Mogibacterium*	0.006	0.001 - 0.013	0.006	0.005 - 0.008	0.747
Firmicutes > Erysipelotrichi > Erysipelotrichales > Erysipelotrichaceae > RFN20	0.008	0.003 - 0.013	0.010	0.008 - 0.015	0.376
Firmicutes > Erysipelotrichi > Erysipelotrichales > Erysipelotrichaceae > p-75-a5	0.004	0.000 - 0.006	0.004	0.002 - 0.008	0.746
Planctomycetes > Planctomycetia > Pirellulales > Pirellulaceae > unclassified	0.001	0.000 - 0.003	0.001	0.000 - 0.002	0.867
Proteobacteria > Alphaproteobacteria > RF32 > unclassified > unclassified	0.000	0.000 - 0.001	0.001	0.000 - 0.001	0.784
Proteobacteria > Betaproteobacteria > Tremblayales > unclassified > unclassified	0.000	0.000 - 0.000	0.000	0.000 - 0.000	0.314
Proteobacteria > Gammaproteobacteria > Aeromonadales > Succinivibrionaceae > unclassified	0.000	0.000 - 0.002	0.000	0.000 - 0.000	0.140
Spirochaetes > Spirochaetes > Spirochaetales > Spirochaetaceae > *Treponema*	0.005	0.004 - 0.006	0.002	0.001 - 0.005	0.060
Synergistetes > Synergistia > Synergistales > Synergistaceae > unclassified	0.000	0.000 - 0.000	0.000	0.000 - 0.000	0.902
Other Genera <1%	0.028	0.022 - 0.029	0.030	0.029 - 0.032	0.170

[a] IQR – Interquartile range

[b] Level of statistical significance after Bonferroni adjustment for multiple comparisons $P = 0.001$

*Differences between Days 0 and 4

C) Bacterial genera in the faeces of Group B horses on Days 0 and 4 (fed Diet P).

**Taxonomic rank within the domain Bacteria** **Phylum > Class > Order > Family > Genus**	**Relative abundances of Group B horses**				
	**Day 0**		**Day 4**		
	**Median**	**IQR[a]**	**Median**	**IQR[a]**	***P* -Value[b]**
Actinobacteria > Coriobacteria > Coriobacteriales > Coriobacteriaceae > unclassified	0.005	0.002 - 0.015	0.006	0.004 - 0.010	0.872
Armatimonadetes > SJA-176 > RB046 > unclassified > unclassified	0.002	0.001 - 0.003	0.002	0.001 - 0.002	0.868
Bacteroidetes > Bacteroidia > Bacteroidales >unclassified > unclassified	0.091	0.080 - 0.132	0.134	0.117 - 0.181	0.128
Bacteroidetes > Bacteroidia > Bacteroidales > BS11 > unclassified	0.004	0.002 - 0.007	0.007	0.004 - 0.012	0.290
Bacteroidetes > Bacteroidia > Bacteroidales > Bacteroidaceae > BF311	0.010	0.009 - 0.014	0.011	0.008 - 0.011	0.566
Bacteroidetes > Bacteroidia > Bacteroidales > Bacteroidaceae > *Bacteroides*	0.001	0.000 - 0.002	0.003	0.002 - 0.003	0.070
Bacteroidetes > Bacteroidia > Bacteroidales > Porphyromonadaceae > *Paludibacter*	0.001	0.000 - 0.001	0.001	0.000 - 0.001	0.859
Bacteroidetes > Bacteroidia > Bacteroidales > Porphyromonadaceae > *Parabacteroides*	0.000	0.000 - 0.000	0.000	0.000 - 0.000	n/p
Bacteroidetes > Bacteroidia > Bacteroidales > Prevotellaceae > unclassified	0.001	0.001 - 0.002	0.000	0.000 - 0.001	0.058
Bacteroidetes > Bacteroidia > Bacteroidales > Prevotellaceae > *Prevotella*	0.027	0.020 - 0.039	0.035	0.026 - 0.048	0.470
Bacteroidetes > Bacteroidia > Bacteroidales > RF16 > unclassified	0.002	0.000 - 0.002	0.001	0.000 - 0.002	0.804
Bacteroidetes > Bacteroidia > Bacteroidales > S24-7 > unclassified	0.003	0.002 - 0.003	0.003	0.003 - 0.006	0.347
Bacteroidetes > Bacteroidia > Bacteroidales > [Paraprevotellaceae] > unclassified	0.010	0.007 - 0.015	0.012	0.011 - 0.016	0.294
Bacteroidetes > Bacteroidia > Bacteroidales > [Paraprevotellaceae] > CF231	0.012	0.008 - 0.022	0.011	0.006 - 0.014	0.421
Bacteroidetes > Bacteroidia > Bacteroidales > [Paraprevotellaceae] > YRC22	0.043	0.037 - 0.067	0.031	0.022 - 0.050	0.173
Bacteroidetes > Bacteroidia > Bacteroidales > [Paraprevotellaceae] > [Prevotella]	0.006	0.001 - 0.010	0.003	0.001 - 0.008	0.685
Cyanobacteria > 4C0d-2 > YS2 > unclassified > unclassified	0.001	0.000 - 0.002	0.002	0.001 - 0.007	0.139
Fibrobacteres > Fibrobacteria > Fibrobacterales > Fibrobacteraceae > *Fibrobacter*	0.005	0.003 - 0.009	0.010	0.008 - 0.017	0.092
Firmicutes > Clostridia > unclassified > unclassified > unclassified	0.000	0.000 - 0.001	0.002	0.001 - 0.002	0.028
Firmicutes > Clostridia > Clostridiales > unclassified > unclassified	0.163	0.149 - 0.181	0.146	0.131 - 0.164	0.128
Firmicutes > Clostridia > Clostridiales > Christensenellaceae > unclassified	0.005	0.004 - 0.006	0.006	0.003 - 0.007	0.746
Firmicutes > Clostridia > Clostridiales > Clostridiaceae > unclassified	0.006	0.004 - 0.008	0.006	0.003 - 0.012	0.809
Firmicutes > Clostridia > Clostridiales > Clostridiaceae > *Clostridium*	0.010	0.006 - 0.014	0.013	0.007 - 0.015	0.687
Firmicutes > Clostridia > Clostridiales > Eubacteriaceae > *Pseudoramibacter_Eubacterium*	0.002	0.000 - 0.004	0.003	0.002 - 0.005	0.464
Firmicutes > Clostridia > Clostridiales > Lachnospiraceae > unclassified	0.188	0.157 - 0.238	0.179	0.167 - 0.208	0.810
Firmicutes > Clostridia > Clostridiales > Lachnospiraceae > *Blautia*	0.012	0.007 - 0.015	0.007	0.005 - 0.009	0.090
Firmicutes > Clostridia > Clostridiales > Lachnospiraceae > *Coprococcus*	0.011	0.008 - 0.011	0.011	0.008 - 0.012	0.684
Firmicutes > Clostridia > Clostridiales > Ruminococcaceae > unclassified	0.281	0.227 - 0.293	0.226	0.158 - 0.290	0.378
Firmicutes > Clostridia > Clostridiales > Ruminococcaceae > *Oscillospira*	0.008	0.006 - 0.008	0.006	0.004 - 0.011	0.520
Firmicutes > Clostridia > Clostridiales > Ruminococcaceae > *Ruminococcus*	0.019	0.015 - 0.030	0.024	0.017 - 0.037	0.575
Firmicutes > Clostridia > Clostridiales > [Mogibacteriaceae] > unclassified	0.011	0.007 - 0.012	0.015	0.012 - 0.016	0.092
Firmicutes > Clostridia > Clostridiales > [Mogibacteriaceae] > *Mogibacterium*	0.005	0.004 - 0.005	0.005	0.002 - 0.009	0.808
Firmicutes > Erysipelotrichi > Erysipelotrichales > Erysipelotrichaceae > RFN20	0.003	0.003 - 0.008	0.002	0.002 - 0.004	0.290
Firmicutes > Erysipelotrichi > Erysipelotrichales > Erysipelotrichaceae > p-75-a5	0.002	0.001 - 0.002	0.001	0.000 - 0.002	0.316
Planctomycetes > Planctomycetia > Pirellulales > Pirellulaceae > unclassified	0.000	0.000 - 0.000	0.001	0.000 - 0.001	0.211
Proteobacteria > Alphaproteobacteria > RF32 > unclassified > unclassified	0.000	0.000 - 0.001	0.000	0.000 - 0.001	0.847
Proteobacteria > Betaproteobacteria > Tremblayales > unclassified > unclassified	0.000	0.000 - 0.001	0.001	0.000 - 0.002	0.162
Proteobacteria > Gammaproteobacteria > Aeromonadales > Succinivibrionaceae > unclassified	0.000	0.000 - 0.000	0.000	0.000 - 0.000	n/p
Spirochaetes > Spirochaetes > Spirochaetales > Spirochaetaceae > *Treponema*	0.002	0.002 - 0.004	0.005	0.003 - 0.007	0.075
Synergistetes > Synergistia > Synergistales > Synergistaceae > unclassified	0.000	0.000 - 0.000	0.000	0.000 - 0.000	0.317
Other Genera <1%	0.033	0.029 - 0.036	0.032	0.026 - 0.038	0.936

[a] IQR – Interquartile range

[b] Level of statistical significance after Bonferroni adjustment for multiple comparisons $P = 0.001$

n/p – no *P*-value
