# Supplementary material for: Faecal Microbiota of Forage-Fed Horses in New Zealand and the Population Dynamics of Microbial Communities following Dietary Change
Source: PLoS One. 2014 Nov 10;9(11):e112846. doi: 10.1371/journal.pone.0112846 (PMC4226576; doi:10.1371/journal.pone.0112846)
Supplement: Table S4 — Comparison of the relative abundances of archaeal taxa (clade-level) in the faeces of Group A and B horses on Days 0 and 4 of the study. A) Group A horses fed two different diets in Day 0 (Diet F) and Day 4 (Diet P). B) Group B horses fed pasture (Diet P) on Days 0 and 4. C) Group A and B horses fed pasture (Diet P) on Day 4. (PDF) [file pone.0112846.s008.pdf]

**Table S4. Comparison of the relative abundances of archaeal taxa (clade-level) in the faeces of Group A and B horses on Days 0 and 4 of the study.**

**A) Group A horses fed two different diets in Day 0 (Diet F) and Day 4 (Diet P)**

| **Taxonomic rank under the domain Archaea** | **Relative abundances of Group A horses** | | | | |
|---|---|---|---|---|---|
| **Phylum > Class > Order > Family > Genus / Clade** | **Day 0** | | **Day 4** | | |
| | **Median** | **IQR**[a] | **Median** | **IQR** | ***P* – Value**[b] |
| Euryarchaeota > Methanobacteria > Methanobacteriales > Methanobacteriaceae > *Methanobrevibacter* > *Methanobrevibacter_ruminantium*_and_relatives | 0.630 | 0.413 - 0.897 | 0.398 | 0.244 - 0.719 | 0.200 |
| Euryarchaeota > Methanomicrobia > Methanomicrobiales > *Genera_incertae_sedis* > *Methanocorpusculum*_and_relatives | 0.177 | 0.056 - 0.309 | 0.591 | 0.253 - 0.722 | 0.109 |
| Euryarchaeota > Thermoplasmata > Thermoplasmatales > Rumen Cluster C_and_relatives | 0.016 | 0.003 - 0.031 | 0.017 | 0.009 - 0.034 | 0.809 |
| Euryarchaeota > Methanobacteria > Methanobacteriales > Methanobacteriaceae > *Methanobrevibacter* > *Methanobrevibacter_gottschalkii*_and_relatives | 0.014 | 0.000 - 0.028 | 0.000 | 0.000 - 0.000 | 0.060 |
| Euryarchaeota > Methanobacteria > Methanobacteriales > Methanobacteriaceae > *Methanosphaera* | 0.011 | 0.003 - 0.025 | 0.000 | 0.000 - 0.000 | 0.016 |
| Other Taxa <1% | 0.000 | 0.000 - 0.000 | 0.000 | 0.000 - 0.000 | 1.000 |

[a] IQR – Interquartile range
[b] Level of statistical significance after Bonferroni adjustment for multiple comparisons $P = 0.008$

**B) Group B horses fed pasture (Diet P) on Days 0 and 4**

| **Taxonomic rank under the domain Archaea** | **Relative abundances of Group B horses** | | | | |
|---|---|---|---|---|---|
| **Phylum > Class > Order > Family > Genus / Clade** | **Day 0** | | **Day 4** | | |
| | **Median** | **IQR**[a] | **Median** | **IQR**[a] | ***P* – Value**[b] |
| Euryarchaeota > Methanobacteria > Methanobacteriales > Methanobacteriaceae > *Methanobrevibacter* > *Methanobrevibacter_ruminantium*_and_relatives | 0.663 | 0.569 - 0.769 | 0.575 | 0.431 - 0.722 | 0.465 |
| Euryarchaeota > Methanomicrobia > Methanomicrobiales > *Genera_incertae_sedis* > *Methanocorpusculum*_and_relatives | 0.319 | 0.219 - 0.431 | 0.395 | 0.253 - 0.569 | 0.465 |
| Euryarchaeota > Thermoplasmata > Thermoplasmatales > Rumen Cluster C_and_relatives | 0.016 | 0.009 - 0.038 | 0.019 | 0.016 - 0.034 | 1.000 |
| Euryarchaeota > Methanobacteria > Methanobacteriales > Methanobacteriaceae > *Methanobrevibacter* > *Methanobrevibacter_gottschalkii*_and_relatives | 0.000 | 0.000 - 0.003 | 0.000 | 0.000 - 0.003 | 0.827 |
| Euryarchaeota > Methanobacteria > Methanobacteriales > Methanobacteriaceae > *Methanosphaera* | 0.000 | 0.000 - 0.000 | 0.000 | 0.000 - 0.000 | 0.273 |
| Other Taxa <1% | 0.000 | 0.000 - 0.000 | 0.002 | 0.000 - 0.003 | 0.081 |

[a] IQR – Interquartile range
[b] Level of statistical significance after Bonferroni adjustment for multiple comparisons $P = 0.008$

**C) Group A and B horses fed pasture (Diet P) on Day 4**

| **Taxonomic rank under the domain Archaea** **Phylum > Class > Order > Family > Genus / Clade** | **Relative abundances on Day 4** | | | | |
|---|---|---|---|---|---|
| | **Group A** | | **Group B** | | |
| | **Median** | **IQR**[a] | **Median** | **IQR**[a] | ***P* – Value**[b] |
| Euryarchaeota > Methanomicrobia > Methanomicrobiales > *Genera_incertae_sedis* > *Methanocorpusculum*_and_relatives | 0.591 | 0.253 - 0.722 | 0.395 | 0.253 - 0.569 | 0.370 |
| Euryarchaeota > Methanobacteria > Methanobacteriales > Methanobacteriaceae > *Methanobrevibacter* > *Methanobrevibacter_ruminantium*_and_relatives | 0.398 | 0.244 - 0.719 | 0.575 | 0.431 - 0.722 | 0.330 |
| Euryarchaeota > Thermoplasmata > Thermoplasmatales > Rumen Cluster C_and_relatives | 0.017 | 0.009 - 0.034 | 0.019 | 0.016 - 0.034 | 0.930 |
| Euryarchaeota > Methanobacteria > Methanobacteriales > Methanobacteriaceae > *Methanobrevibacter* > *Methanobrevibacter_gottschalkii*_and_relatives | 0.000 | 0.000 - 0.000 | 0.000 | 0.000 - 0.003 | 0.670 |
| Euryarchaeota > Methanobacteria > Methanobacteriales > Methanobacteriaceae > *Methanosphaera* | 0.000 | 0.000 - 0.000 | 0.000 | 0.000 - 0.000 | 0.310 |
| Other Taxa <1% | 0.000 | 0.000 - 0.000 | 0.002 | 0.000 - 0.003 | 0.210 |

[a] IQR – Interquartile range
[b] Level of statistical significance after Bonferroni adjustment for multiple comparisons $P = 0.008$
